# Phenolic-Enriched Fractions of *Rubus buergeri* Attenuate LPS-Induced Nitric Oxide Production and Inflammatory Gene Expression in Macrophages

**DOI:** 10.3390/cimb48050507

**Published:** 2026-05-14

**Authors:** Theophilus Bhatti, Hong-Yi Xiang, Jihyun Lee, Ji-Yeong Bae, Jinu Kim

**Affiliations:** 1 Interdisciplinary Graduate Program in Advanced Convergence Technology & Science, Jeju National University, Jeju 63243, Republic of Korea; theophilus@stu.jejunu.ac.kr (T.B.); hongyixiang1508@gmail.com (H.-Y.X.); 2 College of Pharmacy and Jeju Research Institute of Pharmaceutical Sciences, Jeju National University, Jeju 63243, Republic of Korea; jihyunlee@jejunu.ac.kr; 3 Department of Anatomy, College of Medicine, Jeju National University, Jeju 63243, Republic of Korea

**Keywords:** *Rubus buergeri*, phenolic compound, nitric oxide, inflammatory gene expression, RAW 264.7 macrophage, bioactivity-guided fractionation

## Abstract

*Rubus buergeri* Miq., a wild species native to Jeju Island (Republic of Korea), is a relatively understudied plant with potential as a source of bioactive phenolic compounds. This study investigated the phytochemical composition of *R. buergeri* extract (RBE) and evaluated its antioxidant and anti-inflammatory activities using a bioactivity-guided fractionation approach. Antioxidant capacity was assessed by 2,2-diphenyl-1-picrylhydrazyl (DPPH), 2,2′-azino-bis(3-ethylbenzothiazoline-6-sulfonic acid) (ABTS), and ferric reducing antioxidant power assays (FRAP), along with total phenolic content determination, while anti-inflammatory activity was evaluated by measuring nitric oxide (NO) production and inflammatory gene expression in lipopolysaccharide (LPS)-stimulated RAW 264.7 macrophages. RBE exhibited high phenolic content and strong antioxidant activity; its ethyl acetate and n-butanol fractions demonstrated the greatest antioxidant activities and significantly inhibited LPS-induced NO production. Furthermore, RBE suppressed LPS-induced mRNA expression of *Nos2*, *Ptgs2*, *Tnfa*, *Il1b*, and *Il6*, indicating coordinated inhibition of inflammatory responses. Ultra-high-performance liquid chromatography (UHPLC) analysis identified ellagic acid, ethyl gallate, and epicatechin as major phenolic constituents, with ellagic acid and ethyl gallate showing stronger inhibitory effects on NO production and inflammatory gene expression than epicatechin. These findings suggest that the phenolic constituents of *R. buergeri* modulate NO-associated inflammatory responses and support its potential as a source of anti-inflammatory phytochemicals.

## 1. Introduction

Macrophages play a central role in both innate immunity and inflammation by recognizing and eliminating pathogens through pattern recognition receptors, such as Toll-like receptors [1,2]. Upon stimulation with pathogen-associated molecular patterns, particularly lipopolysaccharide (LPS), macrophages undergo rapid activation that initiates intracellular signaling cascades, leading to the generation of reactive oxygen and nitrogen species [3,4]. Among these, nitric oxide (NO), produced by inducible nitric oxide synthase (iNOS), functions as a key effector molecule involved in host defense and inflammatory responses [5,6,7]. Excessive or sustained NO production disrupts redox homeostasis, promotes oxidative stress, and is closely associated with the transcriptional upregulation of pro-inflammatory mediators, including tumor necrosis factor-α, encoded by the *Tnfa* gene [8,9]; interleukin-1β, encoded by the *Il1b* gene [9,10]; and interleukin-6, encoded by the *Il6* gene [11]. Consequently, persistent macrophage activation and elevated NO levels are implicated in chronic inflammatory pathologies such as cardiovascular disease [12,13], chronic kidney disease [14], neurodegenerative disorders [15,16], and cancer [17]. Given the central role of NO at the interface of oxidative stress and inflammation, modulation of NO production and redox balance has been considered a promising strategy for controlling inflammatory responses [18,19]. Although non-steroidal anti-inflammatory drugs are widely used for this purpose, their long-term administration is associated with adverse effects, including gastrointestinal irritation [20,21], nephrotoxicity [22,23], and cardiovascular complications [24]. Accordingly, growing interest has focused on natural products as alternative sources of anti-inflammatory agents.

Plant-derived phenolic compounds are widely recognized as bioactive components with antioxidant and anti-inflammatory properties [25]. In particular, species of the genus *Rubus* (Rosaceae) have been widely utilized in East Asian traditional medicine and ethnobotanical practices, where fruits, leaves, and aerial parts are consumed as decoctions or medicinal foods for the treatment of inflammation-related conditions, gastrointestinal disorders, and metabolic diseases [26,27]. Several *Rubus* species, including *Rubus coreanus* and *Rubus idaeus*, exhibit strong antioxidant and anti-inflammatory properties and are rich in phenolic compounds [28]. The traditional medicinal use of *Rubus* species for inflammatory conditions suggests that their phenolic constituents may contribute to these therapeutic effects. These ethnopharmacological observations have stimulated growing scientific interest in identifying bioactive phytochemicals within *Rubus* plants that may contribute to their reported medicinal benefits. In contrast to the extensively studied *Rubus* species, *Rubus buergeri* Miq. (*R*. *buergeri*), commonly referred to as winter strawberry, remains poorly characterized. This species is native to Jeju Island, Republic of Korea, and represents a relatively understudied member of the genus despite its regional botanical importance. Previous studies have reported antioxidant and anti-inflammatory activities of *R*. *buergeri* extracts [29,30]; however, the specific phytochemical constituents responsible for these activities and their underlying molecular mechanisms remain largely unclear.

In the present study, we investigated the antioxidant and anti-inflammatory activities of extracts and solvent fractions derived from the aerial parts of *R*. *buergeri* in LPS-stimulated RAW 264.7 macrophages, and evaluated phenolic constituents responsible for suppressing nitric oxide production and inflammatory gene expression. We hypothesized that phenolic-enriched fractions of *Rubus buergeri* attenuate LPS-induced inflammatory responses in macrophages by suppressing nitric oxide production and the expression of pro-inflammatory genes.

## 2. Materials and Methods

### 2.1. Plant Material

The aerial parts (leaves, small branches, and floral buds) of *R*. *buergeri* were collected from Jeju Island, Republic of Korea, in August 2022. The plant material was taxonomically identified by Prof. Ji-Yeong Bae (Jeju National University). A voucher specimen (JNUP-2022-24) was deposited in the Herbarium of Jeju National University for future reference. The collected plant materials were immediately cleaned to remove adhering soil particles and surface moisture, then dried using a forced hot air drying system at 65 °C for approximately 4–6 h until complete dehydration was achieved. No genomic or transcriptomic analyses were performed in this study.

### 2.2. Extraction and Fractionation of R. buergeri

For extraction, 1.05 kg of dried and powdered aerial parts of *R*. *buergeri* were immersed in 70% ethanol at room temperature at a solid-to-solvent ratio of 1:10 (*w*/*v*) in a conical flask [31]. The mixture was then subjected to three 90-min sonication cycles (Powersonic 520, Hwashintech, Daegu, Republic of Korea), each cycle comprising three 30-min sonication bursts interspersed with periodic mixing and breaks of more than 1 h between cycles. Following sonication, the material was allowed to settle. The supernatant was decanted and clarified by centrifugation at 4500 rpm for 5 min (Avanti J-15R, Beckman Coulter, Brea, CA, USA) at 4 °C, followed by filtration through Whatman No. 1 filter paper (Maidstone, UK). A rotary evaporator (Heidolph, Schwabach, Germany) was used to concentrate the filtrate under reduced pressure (80 rpm, 50 °C water bath, −8 °C chiller). Any residual moisture was removed by freeze-drying for 3 days. Following this, the total *R*. *buergeri* extract (RBE) underwent sequential partitioning into various fractions using solvents of progressively increasing polarity: hexane, methyl chloride, ethyl acetate, initially n-butanol and subsequently hydrated butanol, and finally water. The total extract and dried fractions were weighed to calculate their respective percentage yields, then stored at 4 °C until the analytical procedures could commence.

### 2.3. Radical Scavenging Assays

For the 2,2-diphenyl-1-picrylhydrazyl (DPPH) assay, a fresh 200 µM DPPH solution was prepared by dissolving 7.8 mg of DPPH (Alfa Aesar, Thermo Fisher Scientific, Ward Hill, MA, USA) in 100 mL of methanol. Samples were serially diluted to concentrations ranging from 3.9 to 250 µg/mL. Butylated hydroxytoluene (Tokyo Chemical Industry, Tokyo, Japan) and L-ascorbic acid (Sigma-Aldrich, St. Louis, MO, USA) were used as positive controls. Samples and DPPH solution were mixed in a 4:1 ratio to achieve an initial absorbance of approximately 0.440 at 517 nm using a Spark multimode microplate reader (Tecan, Männedorf, Switzerland), then incubated for 30 min at room temperature in the dark. DPPH scavenging activity (%) was calculated as [(absorbance of the control (methanol) − absorbance of the sample)/absorbance of the control] × 100. The half-maximal inhibitory concentration (IC_50_) was determined by regression analysis using Microsoft Excel (Microsoft, Redmond, WA, USA). For the 2,2′-azino-bis(3-ethylbenzothiazoline-6-sulfonic acid) (ABTS) assay, the ABTS radical cation was generated by mixing 7 mM ABTS (Roche Diagnostics, Mannheim, Germany) with 2.45 mM potassium persulfate (Sigma-Aldrich) and incubating the mixture in the dark at room temperature overnight. The solution was diluted with ethanol to achieve an absorbance of approximately 0.735 at 734 nm. Trolox (15 mM in ethanol; Tokyo Chemical Industry, Tokyo, Japan) was used as the standard. Samples (0.5 and 1.0 mg/mL) or standards (10 µL) were added to 190 µL of the diluted ABTS solution in 96-well microplates, with ethanol serving as the control. After 6 min of incubation at room temperature in the dark, absorbance was measured at 734 nm using the Spark multimode microplate reader (Tecan), and results were expressed as Trolox equivalent antioxidant concentration (TEAC), calculated from a Trolox standard curve and reported as mM Trolox equivalents per gram of sample. For the ferric reducing antioxidant power (FRAP) assay, freshly prepared FRAP reagent consisted of 10 mM 2,4,6-tris(2-pyridyl)-s-triazine (Sigma-Aldrich) in 40 mM hydrochloric acid, 20 mM iron(III) chloride, and 0.3 M acetate buffer at pH 3.6 in a 10:10:100 (*v*/*v*/*v*) ratio, pre-warmed to 37 °C. Samples were mixed with the reagent at a 1:9 ratio. To ensure reaction completion under experimental conditions, absorbance was measured after 24 h at 593 nm using the Spark multimode microplate reader (Tecan), and the reducing power was expressed as TEAC (mM/g), calculated from a Trolox standard curve.

### 2.4. Total Phenolic and Flavonoid Contents

The total phenolic content (TPC) was determined using the Folin–Ciocalteu colorimetric method. Gallic acid (Tokyo Chemical Industry) was used as the standard to generate a standard curve with concentrations ranging from 12.5 to 200 µg/mL in methanol. For each sample, 100 µL of the sample or standard, 500 µL of 10% Folin–Ciocalteu reagent (diluted 1:10 with deionized water), and 400 µL of sodium carbonate solution (10.125 g dissolved in 50 mL of distilled water) were added to an Eppendorf tube. After incubation, the absorbance of the reaction mixture was measured at 765 nm using the Spark multimode microplate reader (Tecan). Results were expressed as milligrams of gallic acid equivalents (GAE) per gram of dry weight, calculated from the linear regression equation of the standard curve. The total flavonoid content (TFC) was determined using the aluminum chloride colorimetric method. Quercetin (Sigma-Aldrich) was used as the standard to generate a standard curve with concentrations ranging from 10 to 1000 µg/mL in methanol. For each sample, 100 µL of the sample or standard, 300 µL of 95% ethanol, 560 µL of distilled water, 20 µL of 10% aluminum chloride in distilled water, and 20 µL of 1 M potassium acetate in distilled water were added to an Eppendorf tube. After incubation, the absorbance of the reaction mixture was measured at 415 nm using the Spark multimode microplate reader (Tecan). Results were expressed as milligrams of quercetin equivalents (QE) per gram of dry weight.

### 2.5. Ultra-High-Performance Liquid Chromatography (UHPLC) Analysis of Major Phenolic Constituents

The major phenolic constituents of RBE and its active fractions were analyzed using an Agilent 1260 Infinity II UHPLC system (Agilent, Santa Clara, CA, USA) equipped with UV detection set at 280 nm [31]. Chromatographic separation was performed on an Agilent Poroshell 120 EC-C18 (Agilent, Santa Clara, CA, USA) column (100 × 4.6 mm, 2.7 µm) maintained at 40 °C. The mobile phase consisted of solvent A (0.1% formic acid in water) and solvent B (0.1% formic acid in acetonitrile), with the following gradient program: 1% B (0–1 min), 1–5% B (1–5 min), 5–20% B (5–10 min), 20–40% B (10–15 min), 40–50% B (15–20 min), and 50–85% B (20–30 min), followed by re-equilibration to the conditions. The flow rate was set at 0.8 mL/min, and the injection volume was 5 µL. Ellagic acid, ethyl gallate, and epicatechin were tentatively identified by comparing their retention times and UV spectra with those of authentic standards.

### 2.6. Cell Culture and Exposure to LPS

The murine macrophage cell line RAW 264.7 (Korean Cell Line Bank, Seoul, Republic of Korea), originally obtained from the American Type Culture Collection (product no. TIB-71), was maintained in a humidified incubator at 37 °C with 5% CO_2_. Cells were cultured in Dulbecco’s Modified Eagle Medium (DMEM; Thermo Fisher Scientific, Grand Island, NY, USA) supplemented with 10% heat-inactivated fetal bovine serum and 1% penicillin-streptomycin solution (100 units/mL penicillin and 100 µg/mL streptomycin; Thermo Fisher Scientific), as described previously [32]. Cells were passaged every 1–2 days using a cell scraper after reaching 80–90% confluence, followed by centrifugation and resuspension in fresh culture medium. The RBE, ethyl acetate fraction, and n-butanol fraction were dissolved in dimethyl sulfoxide (DMSO) to prepare stock solutions at a concentration of 100 mg/mL. Ellagic acid (Sigma-Aldrich), ethyl gallate (Tokyo Chemical Industry), epicatechin (Tokyo Chemical Industry), diethylamine NONOate diethylammonium salt (DEA NONOate, Sigma-Aldrich), and S-nitrosoglutathione (GSNO, Sigma-Aldrich) were dissolved in DMSO to prepare stock solutions at 10 mM. The final concentration of DMSO did not exceed 0.1% (*v*/*v*) in any experimental condition. The concentration ranges of RBE, its fractions, phenolic compounds, and NO donors were determined based on preliminary experiments assessing cell viability and biological responses. Cells were treated with various concentrations of RBE, ethyl acetate fraction, n-butanol fraction, ellagic acid, ethyl gallate, epicatechin, DEA NONOate, and GSNO in DMEM for 3 h, followed by 18 or 24 h in the presence or absence of 1 µg/mL LPS derived from *Escherichia coli* O111:B4 (InvivoGen, San Diego, CA, USA). For basal control experiments, cells were treated with RBE, ethyl acetate fraction, n-butanol fraction, ellagic acid, ethyl gallate, and epicatechin for 3 h without LPS stimulation.

### 2.7. Cell Viability

The cytotoxic effects of the samples were evaluated using the 3-(4,5-dimethylthiazol-2-yl)-2,5-diphenyltetrazolium bromide (MTT) colorimetric assay, as described previously [33]. After treatment in a 96-well plate, 20 µL of MTT solution (Gibco BRL, Rockville, MD, USA) was added to each well, and the plates were incubated at 37 °C for 2 h to allow for formazan crystal formation. The medium was then carefully removed, and the insoluble formazan crystals were dissolved by adding 200 µL of DMSO with thorough mixing. Absorbance was measured at 540 nm using a SpectraMax i3x multi-mode microplate reader (Molecular Devices, San Jose, CA, USA) in the Bio-Health Materials Core-Facility at Jeju National University. When necessary, the dissolved formazan solutions were diluted with DMSO at a 1:4 ratio, and the absorbance values were adjusted accordingly. Cell viability (%) was calculated as (absorbance of treated cells/absorbance of control cells) × 100.

### 2.8. NO Production

The inhibitory effect of the samples on NO production was evaluated by measuring the concentration of nitrite, a stable NO metabolite, in the cell culture supernatant using the Griess reagent (DogenBio, Seoul, Republic of Korea). Positive controls (LPS-stimulated, vehicle-treated cells) and negative controls (unstimulated cells) were included. After treatment in a 24-well plate, 100 μL of cell culture media from each well was transferred to a 96-well plate and mixed with 100 μL of Griess reagent (reagents A and B). The plate was incubated in the dark at room temperature for 10 min after the addition of each reagent. Absorbance was then measured at 540 nm using a Spark multimode microplate reader (Tecan). NO production was quantified as nitrite concentration (µM) using a sodium nitrite standard curve.

### 2.9. Bioinformatics Analysis of Gene Expression Profiles in LPS-Exposed RAW 264.7 Macrophages

For the identification of differentially expressed genes, the gene expression dataset GSE76562, based on the GPL18802 [MoGene-2_0-st] Affymetrix Mouse Gene 2.0 ST Array [mogene20st_Mm_ENTREZG_18.0.0] platform, was downloaded from the National Center for Biotechnology Information Gene Expression Omnibus (GEO) database. Differential gene expression analysis was conducted between control and LPS-treated (10 ng/mL for 6 h) RAW 264.7 macrophages (*n* = 3). Genes with a log_2_ fold change > 1 and a false discovery rate (FDR)-adjusted *p* < 0.05 were considered significantly upregulated, resulting in 688 genes identified using the GEO2R tool (https://www.ncbi.nlm.nih.gov/geo/geo2r/, accessed on 3 October 2025). The Benjamini–Hochberg method was applied to correct for multiple testing. For enrichment analysis, these 688 upregulated genes were analyzed for Gene Ontology (GO) Biological Process enrichment analysis using the Database for Annotation, Visualization and Integrated Discovery (DAVID) tool (https://davidbioinformatics.nih.gov/, accessed on 3 October 2025). In the enrichment output, gene count, FDR-adjusted *p* value, and gene ratio (calculated as the number of genes associated with a specific GO term divided by the total number of input genes) were used to evaluate the significance and representation of each biological process. The GO term “cellular response to lipopolysaccharide”, which includes 49 genes, was selected for further analysis. To explore protein–protein interactions, a network was constructed using the Search Tool for the Retrieval of Interacting Genes (STRING) database (default confidence score), and key topological parameters, including degree, betweenness centrality, closeness centrality, stress, and neighborhood connectivity, were evaluated. Hub gene analysis was conducted using the cytoHubba (v0.1) plugin in Cytoscape (v3.10.4), applying the Maximal Clique Centrality (MCC) algorithm. The top 10 hub genes were identified as the most central regulators within the LPS-responsive network.

### 2.10. RNA Isolation and Quantitative Reverse Transcription-Polymerase Chain Reaction (qRT-PCR)

After treatment in 60 mm culture dishes, total RNA from RAW 264.7 cells was isolated using the TRIzol-chloroform method, as described previously [34]. RNA concentration and purity were measured using a Nanodrop One Microvolume UV-Vis spectrophotometer (Thermo Fisher Scientific), and the A260/A280 ratio was used to assess RNA quality. Only samples with ratios between 1.7 and 2.0 were used for further analysis. First-strand complementary DNA (cDNA) was synthesized from 1.5 μg of total RNA. To perform qRT-PCR, Bio-Rad iTaq Universal SYBR Green Supermix (Bio-Rad Laboratories, Hercules, CA, USA) was used. Various pro-inflammatory biomarkers were analyzed for their mRNA expression using the Bio-Rad CFX Connect Real-Time System (Bio-Rad Laboratories). The amplification conditions were as follows: initial denaturation at 95 °C for 3 min, followed by 40 cycles of denaturation at 95 °C for 5 s and annealing/extension at 60 °C for 30 s. Melt-curve analysis was performed to confirm amplification specificity. Relative mRNA expression levels were calculated using the 2^−ΔΔCt^ method, with *Gapdh* used as the internal reference gene. Primer sequences are listed in Table 1.

### 2.11. Statistical Analysis

Numerical data were analyzed using SigmaPlot 14.0 (Systat Software, San Jose, CA, USA), as previously described [33]. Prior to comparing differences among groups, data normality was assessed using the Shapiro–Wilk test, and homogeneity of variance was evaluated using the Brown-Forsythe test. When assumptions were not met, logarithmic transformation was applied. Parametric data were analyzed using one-way or two-way analysis of variance (ANOVA). When heteroscedasticity was detected, Welch’s ANOVA or linear models with heteroscedasticity-consistent (HC3) standard errors were applied. Post hoc multiple comparisons were performed using Holm–Sidak’s test, Games-Howell test, or Holm-corrected Welch’s *t*-tests, as appropriate for the experimental design and variance structure. Non-parametric data were analyzed using the Kruskal–Wallis test followed by Dunn’s post hoc test. Parametric data are presented as mean ± standard error of the mean (SEM) with individual data points, while non-parametric data are presented as medians with interquartile ranges. For correlation analyses between NO production and mRNA expression, data normality was assessed using the Shapiro–Wilk test. Parametric correlations were analyzed using Pearson’s correlation coefficient (*r*), whereas non-parametric correlations were assessed using Spearman’s rank correlation coefficient (*ρ*). A *p* < 0.05 was considered statistically significant. All raw data and corresponding statistical results are provided in the Supplementary File S1.

## 3. Results

### 3.1. Antioxidant Activity of Rubus buergeri Extract and Its Solvent Fractions

Sequential fractionation of the 70% ethanol extract of *R. buergeri* aerial parts yielded 77.5 g of crude extract (7.75%). This extract was further partitioned into hexane (lower layer, 20.55 g, 26.52%; upper layer, 2.11 g, 2.72%), methylene chloride (0.58 g, 0.75%), ethyl acetate (2.45 g, 3.16%), n-butanol (5.12 g, 6.60%), hydrated butanol (1.58 g, 2.04%), and water (22.26 g, 28.72%) fractions. The antioxidant potential of RBE and its solvent fractions was evaluated using DPPH, ABTS, and FRAP assays. In the DPPH assay (Table 2), L-ascorbic acid and butylated hydroxytoluene (BHT) served as positive controls. The crude RBE demonstrated substantial free radical scavenging activity, with a median IC_50_ of 35.3 µg/mL (interquartile range (IQR), 34.7–35.4). Among RBE and its solvent fractions, the ethyl acetate and n-butanol fractions exhibited the lowest IC_50_ values, with significant differences among groups confirmed by the Kruskal–Wallis test (*H* = 22.680, *p* = 0.002). In the ABTS assay (Table 3), the crude extract showed a mean TEAC value of 2.23 ± 0.02 mM/g. The ethyl acetate fraction exhibited the highest antioxidant capacity, followed by the n-butanol fraction, with significant differences among RBE and its solvent fractions determined by Welch’s ANOVA (*F* = 2736.23, *p* < 0.001). Similarly, in the FRAP assay (Table 4), the crude extract exhibited a median TEAC value of 263.4 mM/g (IQR, 256.7–272.4), while the ethyl acetate fraction demonstrated the highest ferric reducing antioxidant power, followed by the n-butanol fraction. Significant differences among RBE and its solvent fractions were confirmed by the Kruskal–Wallis test (*H* = 46.288, *p* ≤ 0.001). These results indicate that the ethyl acetate and n-butanol fractions exhibit the strongest antioxidant activities among the tested RBE-derived samples.

### 3.2. Phytochemical Composition of RBE and Its Fractions and Their Correlation with Antioxidant Activity

**Figure 1:**
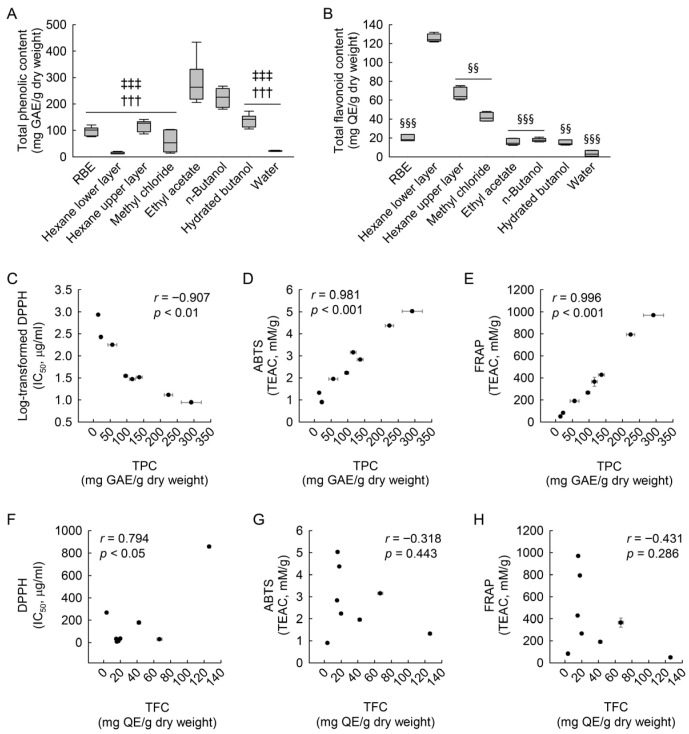
Total phenolic and total flavonoid content of RBE and its fractions, and their correlations with antioxidant activities. (**A**) TPC is expressed as mg of GAE per gram of dry weight for RBE and its fractions (*n* = 7). ††† *p* < 0.001 versus ethyl acetate fraction, ‡‡‡ *p* < 0.001 versus n-butanol fraction. (**B**) TFC is expressed as milligrams of QE per gram of dry weight (*n* = 5). (**A**,**B**) Statistical significance was determined by the Kruskal–Wallis test followed by Dunn’s post hoc test. §§ *p* < 0.01, §§§ *p* < 0.001 versus hexane lower layer fraction. Correlations between TPC and antioxidant activities of DPPH (**C**), ABTS (**D**), FRAP (**E**) or between TFC and those of DPPH (**F**), ABTS (**G**), FRAP (**H**) were analyzed (*n* = 8 independent extract/fraction samples). Statistical correlations were determined using Pearson’s correlation analysis, yielding the correlation coefficient *r*.

To characterize the phytochemical composition of RBE and its solvent fractions, the TPC and TFC were quantified. The crude RBE contained a median TPC of 101.1 mg GAE/g (IQR, 83.6–106.1), and a median TFC of 18.0 mg QE/g (IQR, 17.1–23.6). Following fractionation, the ethyl acetate and n-butanol fractions exhibited the highest TPC (Figure 1A), with significant differences among RBE and its solvent fractions confirmed by the Kruskal–Wallis test (*H* = 50.098, *p* ≤ 0.001). For TFC, significant differences among fractions were also observed (Figure 1B; *H* = 35.017, *p* ≤ 0.001). Correlation analysis revealed that TPC was strongly associated with antioxidant activities, showing significant correlation with log-transformed DPPH scavenging activity (*r* = −0.907, *p* < 0.01; Figure 1C), ABTS (*r* = 0.981, *p* < 0.001; Figure 1D), and FRAP (*r* = 0.996, *p* < 0.001; Figure 1E). TFC was significantly correlated with DPPH activity (*r* = 0.794, *p* < 0.05; Figure 1F), but not with ABTS (*r* = 0.318, *p* = 0.443; Figure 1G), or FRAP (*r* = 0.431, *p* = 0.286; Figure 1H). These results indicate that phenolic compounds are enriched in the ethyl acetate and n-butanol fractions and are closely associated with their antioxidant activities.

### 3.3. Effects of RBE and Its Fractions on Macrophage Viability and LPS-Induced NO Production

**Figure 2:**
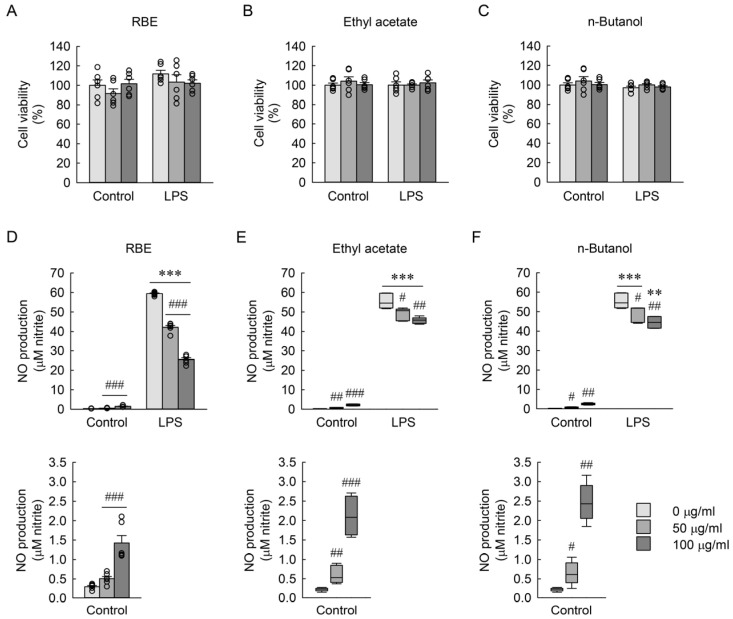
Effects of RBE and its ethyl acetate and n-butanol fractions on cell viability and NO production in macrophages exposed to LPS. RAW 264.7 macrophages were treated with RBE, ethyl acetate fraction, or n-butanol fraction at concentrations of 0, 50, and 100 µg/mL for 3 h prior to exposure to 1 µg/mL LPS for 24 h (*n* = 6). Cell viability of RBE (**A**), ethyl acetate fraction (**B**), and n-butanol fraction (**C**). Statistical significance was determined by two-way ANOVA followed by Holm–Sidak’s post hoc test (parametric data). NO production of RBE (**D**), ethyl acetate fraction (**E**), and n-butanol fraction (**F**). The control group with lower values is additionally shown in the graph below to improve visualization of basal NO production due to scale differences. A modest increase in basal NO production was observed under control conditions. Statistical significance was determined by two-way ANOVA followed by Holm–Sidak’s post hoc test for RBE (**D**), Welch’s *t*-tests with HC3 standard errors with Holm correction for the ethyl acetate fraction (**E**), and the Kruskal–Wallis test followed by Dunn’s post hoc test for the n-butanol fraction (**F**). ** *p* < 0.01, *** *p* < 0.001 versus control; # *p* < 0.05, ## *p* < 0.01, ### *p* < 0.001 versus 0 µg/mL.

Based on these findings, we next investigated whether these phenolic-enriched fractions influence macrophage viability and LPS-induced nitric oxide production. The potential cytotoxicity of RBE and its ethyl acetate and n-butanol fractions in macrophages was evaluated using the MTT assay with and without LPS exposure. As shown in Figure 2A–C, treatment with RBE and its fractions did not significantly affect macrophage viability under any experimental condition. Two-way ANOVA confirmed no significant effects of LPS, dose, or their interaction for RBE (LPS: *F* = 3.762, *p* = 0.062; dose: *F* = 1.360, *p* = 0.272; interaction: *F* = 0.805, *p* = 0.456), the ethyl acetate fraction (LPS: *F* = 0.070, *p* = 0.794; dose: *F* = 0.269, *p* = 0.766; interaction: *F* = 0.574, *p* = 0.569), or the n-butanol fraction (LPS: *F* = 2.529, *p* = 0.122; dose: *F* = 1.199, *p* = 0.316; interaction: *F* = 0.061, *p* = 0.941).

The effects of the extract and its fractions on LPS-induced NO production were then examined. Exposure to LPS markedly increased NO levels compared to control cells (Figure 2D–F). Pretreatment with RBE significantly attenuated this increase in a dose-dependent manner (Figure 2D), whereas the ethyl acetate and n-butanol fractions also reduced NO production, without a clear dose-dependent trend (Figure 2E,F). In LPS-unexposed control cells, treatment with RBE, the ethyl acetate fraction, and the n-butanol fraction resulted in a statistically significant and dose-dependent increase in basal NO levels. Two-way ANOVA revealed significant main effects of LPS and dose, as well as a significant interaction, for RBE (LPS: *F* = 3627.845, *p* < 0.001; dose: *F* = 8.809, *p* < 0.001; interaction: *F* = 98.773, *p* < 0.001). For the ethyl acetate fraction, a two-factor linear model with HC3 standard errors revealed significant main effects of LPS and dose, as well as a significant interaction (LPS: *F* = 2344.34, *p* < 0.001; dose: *F* = 98.60, *p* < 0.001; interaction: *F* = 110.16, *p* < 0.001). For the n-butanol fraction, the Kruskal–Wallis test indicated significant differences among doses in both control and LPS-stimulated conditions (*H* = 27.168, *p* ≤ 0.001). Together, these results indicate that RBE and its active solvent fractions do not impair macrophage viability and significantly suppress LPS-induced NO production, while modestly increasing basal NO levels under non-stimulated conditions.

### 3.4. Bioinformatics Analysis of LPS-Induced Inflammatory Genes in Macrophages

**Figure 3:**
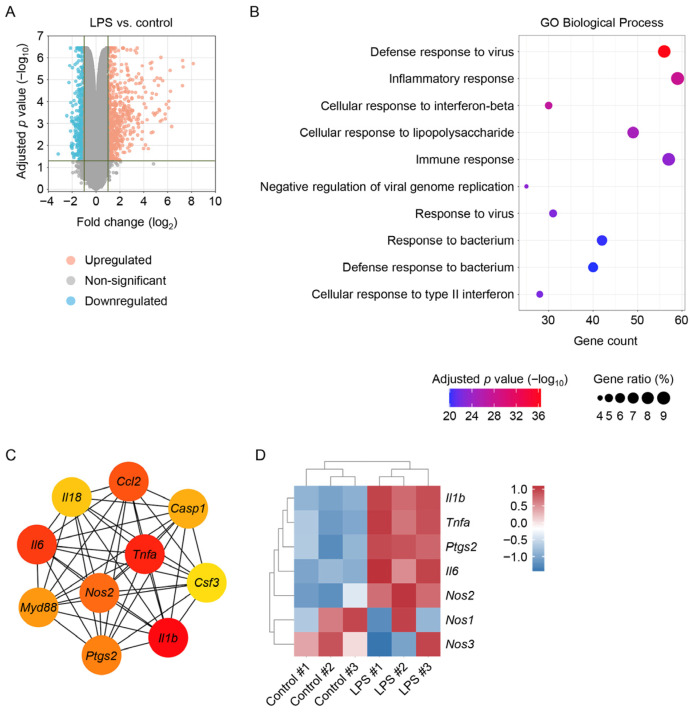
Bioinformatics analysis of LPS-exposed macrophages. (**A**) Volcano plot generated using Bioinformatics.com.cn, illustrating differentially expressed genes between LPS-exposed and control RAW 264.7 macrophages. Genes were classified as upregulated (log_2_ fold change > 1 and false discovery rate (FDR)-adjusted *p* value < 0.05), downregulated (log_2_ fold change < −1 and FDR-adjusted *p* value < 0.05), or non-significant based on the defined thresholds. (**B**) GO Biological Process enrichment analysis of upregulated genes using the Database for Annotation, Visualization and Integrated Discovery (DAVID). The top enriched GO terms are visualized as a bubble chart generated in Bioinformatics.com.cn, with color representing the −log_10_(FDR-adjusted *p*) (Benjamini–Hochberg correction) and circle size indicating the gene ratio (%). (**C**) Protein–protein interaction (PPI) network of the top 10 hub genes, visualized in Cytoscape and identified using the MCC algorithm in the cytoHubba plugin. (**D**) Heatmap showing the expression profiles of the 10 hub genes along with *Nos1* and *Nos3* in control and LPS-exposed groups (*n* = 3 per group), visualized using the OmicShare online platform (https://www.omicshare.com/, accssed on 3 October 2025). The color scale represents z-score–normalized expression levels.

To characterize the transcriptional responses associated with LPS stimulation in macrophages, bioinformatics analysis was conducted using a publicly available microarray dataset (GSE76562) derived from LPS-exposed RAW 264.7 macrophages. As shown in Figure 3A, differential gene expression analysis identified 688 upregulated genes (log_2_ fold change > 1, adjusted *p* < 0.05) following LPS exposure compared to the control. Gene Ontology (GO) Biological Process enrichment analysis revealed significant enrichment of inflammation-related biological processes (Figure 3B). Among these, the GO term “cellular response to lipopolysaccharide” was selected for further analysis, encompassing 49 genes associated with LPS-responsive macrophage activation (Table 5). A protein–protein interaction network constructed from these 49 genes identified 10 hub genes (*Il1b*, *Tnfa*, *Ptgs2*, *Il6*, *Nos2*, *Ccl2*, *Csf3*, *Casp1*, *Myd88*, and *Il18*) as central nodes, determined using the Maximal Clique Centrality (MCC) algorithm implemented in the cytoHubba plugin (Figure 3C). Heatmap visualization further demonstrated that LPS exposure markedly increased the expression of these hub genes, whereas *Nos1* and *Nos3* did not exhibit comparable changes (Figure 3D). These results indicate that LPS stimulation robustly induces a coordinated inflammatory gene network in macrophages.

### 3.5. Effects of RBE on LPS-Induced mRNA Expression of Pro-Inflammatory Markers in Macrophages

**Figure 4:**
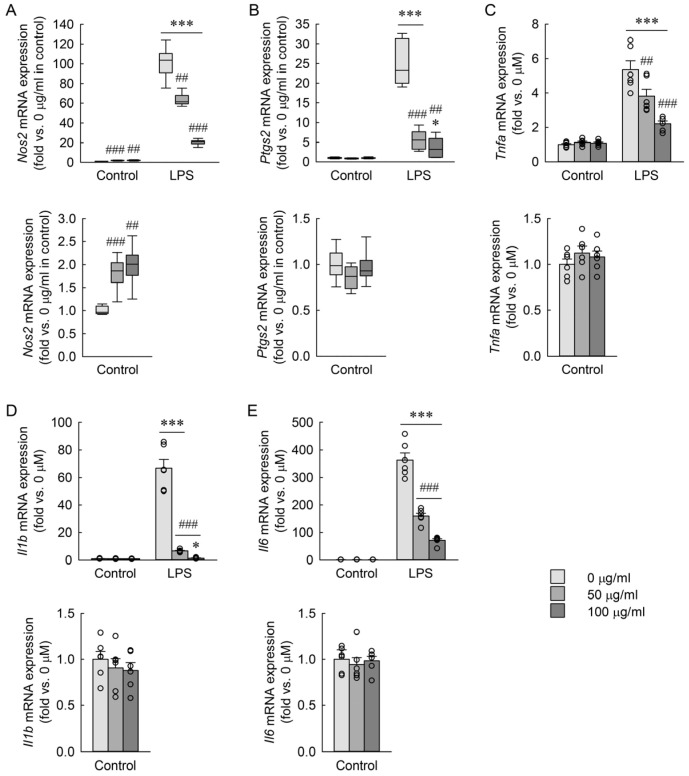
Effects of RBE on mRNA expression of inflammatory markers in macrophages exposed to LPS. RAW 264.7 macrophages were treated with RBE at concentrations of 0, 50, and 100 µg/mL for 3 h, followed by exposure to 1 µg/mL LPS for 18 h (*n* = 6). Data were normalized to the reference gene *Gapdh*. mRNA expressions of *Nos2* (**A**), *Ptgs2* (**B**), *Tnfa* (**C**), *Il1b* (**D**), and *Il6* (**E**) were measured by quantitative reverse transcription-polymerase chain reaction (qRT-PCR). The control group with lower values is additionally displayed in the graph below to improve visualization due to scale differences. Statistical significance was determined using the Kruskal–Wallis test followed by Dunn’s post hoc test (**A**), Welch’s *t*-tests with HC3 standard errors with Holm correction for multiple comparisons (**B**), and two-way ANOVA followed by Holm–Sidak’s post hoc test (**C**–**E**). * *p* < 0.05, *** *p* < 0.001 versus control; ## *p* < 0.01, ### *p* < 0.001 versus 0 µg/mL.

To examine the effects of RBE on the mRNA expression of key inflammatory mediators, RAW 264.7 macrophages were exposed to LPS in the presence or absence of RBE. As shown in Figure 4A–E, LPS markedly increased the expression of *Nos2*, *Ptgs2*, *Tnfa*, *Il1b*, and *Il6* compared with control cells. Pretreatment with RBE significantly suppressed the LPS-induced upregulation of these genes. Among them, *Nos2*, *Tnfa*, *Il1b*, and *Il6* were downregulated in a dose-dependent manner, whereas *Ptgs2* exhibited a non-linear inhibitory pattern. In LPS-unexposed control cells, RBE treatment resulted in a modest but statistically significant increase in *Nos2* expression, while the expression levels of the other genes remained unchanged. The Kruskal–Wallis test revealed significant differences among doses for *Nos2* across all experimental conditions (*H* = 33.228, *p* ≤ 0.001). For *Ptgs2*, a two-factor linear model with HC3 standard errors revealed a significant main effect of LPS and a significant LPS × dose interaction, but no significant main effect of dose (LPS: *F* = 688.90, *p* < 0.001; dose: *F* = 1.19, *p* = 0.317; interaction: *F* = 26.78, *p* < 0.001). Two-way ANOVA demonstrated significant main effects of LPS exposure, RBE dose, and their interaction for *Tnfa* (LPS: *F* = 354.271, *p* < 0.001; dose: *F* = 14.044, *p* < 0.001; interaction: *F* = 19.919, *p* < 0.001), *Il1b* (LPS: *F* = 657.452, *p* < 0.001; dose: *F* = 190.192, *p* < 0.001; interaction: *F* = 165.442, *p* < 0.001), and *Il6* (LPS: *F* = 7323.597, *p* < 0.001; dose: *F* = 64.654, *p* < 0.001; interaction: *F* = 62.278, *p* < 0.001). These results indicate that RBE suppresses LPS-induced transcriptional activation of *Nos2*, *Tnfa*, *Il1b*, and *Il6* in a dose-dependent manner, while *Ptgs2* exhibits a non-linear response to RBE treatment.

### 3.6. Effects of Major Phenolic Compounds of RBE on Macrophage Viability and LPS-Induced NO Production

**Figure 5:**
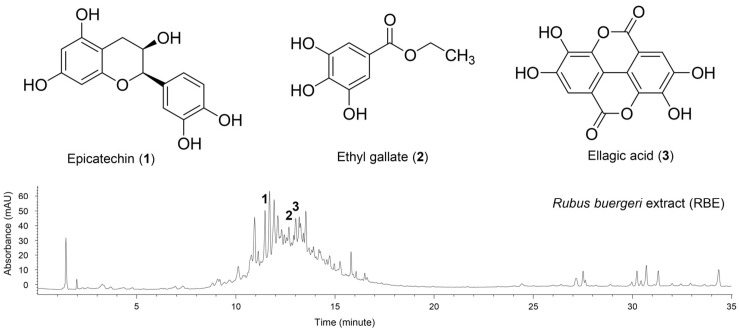
UV chromatograms of RBE in 70% ethanol at 280 nm showing three tentatively assigned major phenolic constituents. Epicatechin (**1**), ethyl gallate (**2**), ellagic acid (**3**).

**Figure 6:**
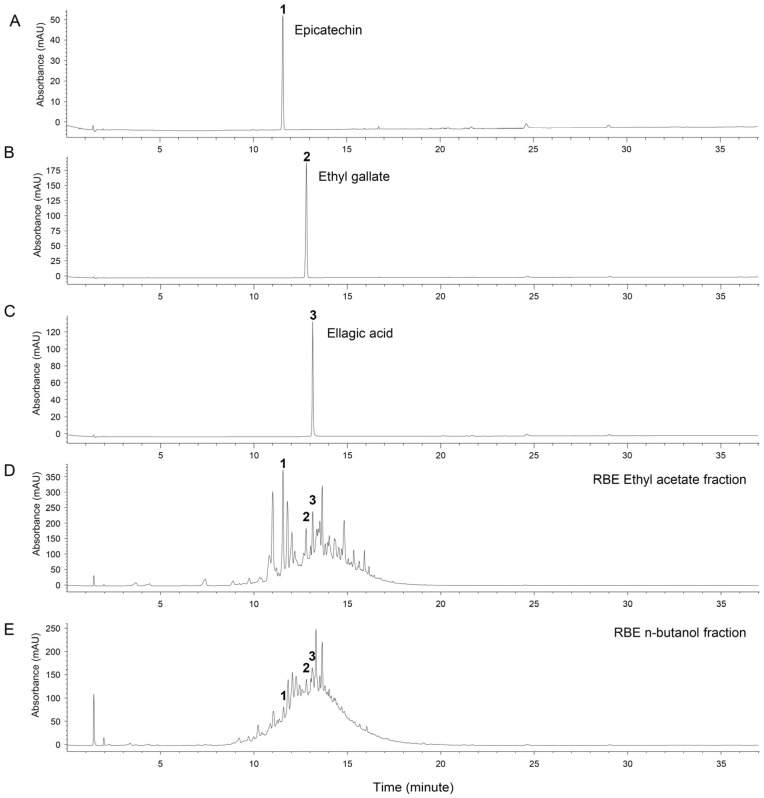
UV chromatograms of ethyl acetate and n-butanol fractions of RBE in 70% ethanol at 280 nm. (**A**) Epicatechin standard, (**B**) ethyl gallate standard, (**C**) ellagic acid standard, (**D**) ethyl acetate fraction, (**E**) n-butanol fraction.

**Figure 7:**
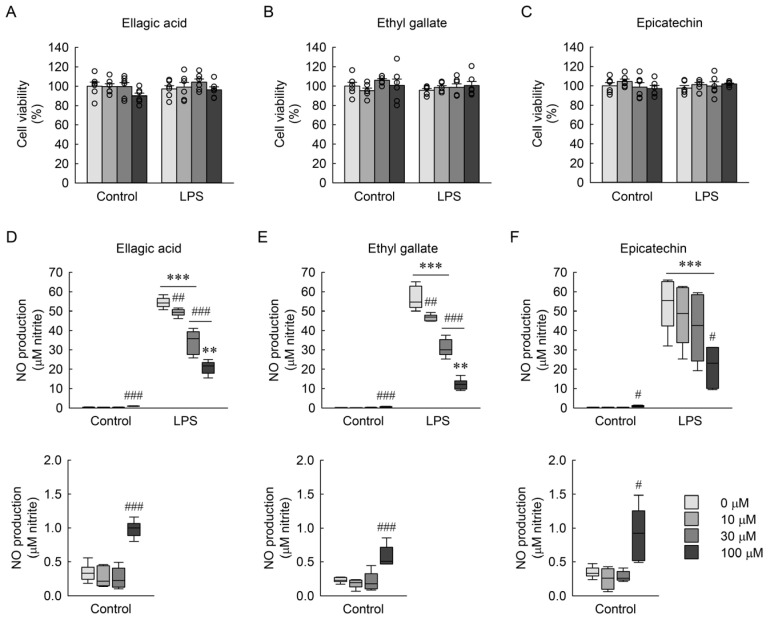
Effects of phytochemical compounds on cell viability and NO production in macrophages exposed to LPS. RAW 264.7 macrophages were treated with ellagic acid, ethyl gallate, and epicatechin at concentrations of 0, 10, 30, and 100 µM for 3 h, followed by exposure to 1 µg/mL LPS for 24 h (*n* = 6). Cell viability of ellagic acid (**A**), ethyl gallate (**B**), and epicatechin (**C**). Statistical significance was determined using two-way ANOVA followed by Holm–Sidak’s post hoc test. NO production of ellagic acid (**D**), ethyl gallate (**E**), and epicatechin (**F**). The control group with lower values is additionally shown in the graph below to improve visualization due to scale differences. Statistical significance was determined using the Kruskal–Wallis test followed by Dunn’s post hoc test for ellagic acid (**D**) and ethyl gallate (**E**) and a two-factor linear model with HC3 standard errors followed by Holm-corrected pairwise comparisons for epicatechin (**F**). ** *p* < 0.01, *** *p* < 0.001 versus control; # *p* < 0.05, ## *p* < 0.01, ### *p* < 0.001 versus 0 µM.

Three major phenolic compounds—ellagic acid, ethyl gallate, and epicatechin—were tentatively identified in the ethyl acetate and n-butanol fractions by UV chromatographic analysis at 280 nm. Peak assignments for these compounds in the UV chromatograms of RBE and its active fractions are shown in Figure 5 and Figure 6. To evaluate their contributions to the anti-inflammatory activity of RBE, their effects on macrophage viability and NO production were examined. None of the compounds exhibited cytotoxic effects up to 100 µM (Figure 7A–C). Two-way ANOVA revealed no significant effects of LPS exposure, compound dose, or their interaction on cell viability for ellagic acid (LPS: *F* = 0.438, *p* = 0.512; dose: *F* = 1.748, *p* = 0.173; interaction: *F* = 0.577, *p* = 0.634), ethyl gallate (LPS: *F* = 0.585, *p* = 0.449; dose: *F* = 0.806, *p* = 0.498; interaction: *F* = 0.700, *p* = 0.558), or epicatechin (LPS: *F* = 0.00295, *p* = 0.957; dose: *F* = 0.719, *p* = 0.546; interaction: *F* = 0.681, *p* = 0.569). Both ellagic acid and ethyl gallate significantly suppressed LPS-induced NO production at all tested concentrations, whereas epicatechin reduced NO levels only at 100 µM (Figure 7D–F). In LPS-unexposed control cells, all three compounds induced a modest but significant increase in basal NO levels at 100 µM. The Kruskal–Wallis test revealed significant differences in NO production among treatment groups for ellagic acid and ethyl gallate (ellagic acid: *H* = 44.224, *p* ≤ 0.001; ethyl gallate: *H* = 44.334, *p* ≤ 0.001). For epicatechin, a two-factor linear model with HC3 standard errors revealed significant main effects of LPS exposure and compound dose, as well as a significant LPS × dose interaction (LPS: *F* = 977.44, *p* < 0.001; dose: *F* = 8.37, *p* < 0.001; interaction: *F* = 10.40, *p* < 0.001). These results indicate that ellagic acid and ethyl gallate robustly suppress LPS-induced NO production, whereas epicatechin exhibits weaker inhibitory activity that is evident only at higher concentrations.

### 3.7. Effects of Major Phenolic Compounds of RBE on LPS-Induced Nos2 mRNA Expression in Macrophages

**Figure 8:**
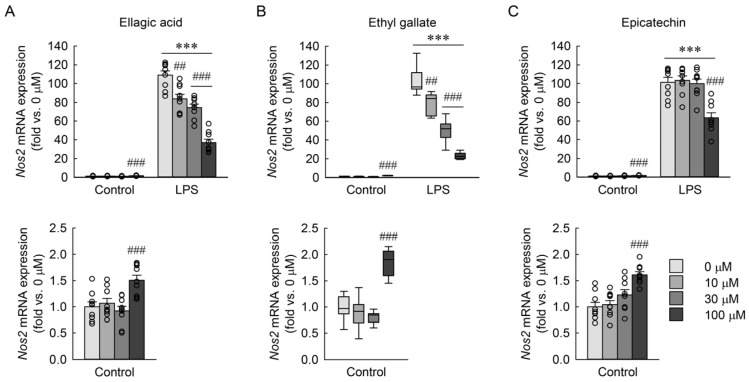
Effects of phytochemical compounds on *Nos2* mRNA expression in macrophages exposed to LPS. RAW 264.7 macrophages were treated with ellagic acid, ethyl gallate, and epicatechin at concentrations of 0, 10, 30, and 100 µM for 3 h, followed by exposure to 1 µg/mL LPS for 18 h (*n* = 9). *Nos2* mRNA expressions of ellagic acid (**A**), ethyl gallate (**B**), and epicatechin (**C**) were measured by quantitative reverse transcription-polymerase chain reaction (qRT-PCR). The control group with lower values is additionally shown in the graph below to improve visualization due to scale differences. Statistical significance was determined using two-way ANOVA followed by Holm–Sidak’s post hoc test for ellagic acid (**A**) and epicatechin (**C**) and the Kruskal–Wallis test followed by Dunn’s post hoc test for ethyl gallate (**B**). *** *p* < 0.001 versus control; ## *p* < 0.01, ### *p* < 0.001 versus 0 µM.

As shown in Figure 8, exposure to LPS markedly increased *Nos2* mRNA expression compared to control cells. In LPS-exposed macrophages, ellagic acid significantly reduced *Nos2* expression at all tested concentrations, although the inhibitory effect was not dose-dependent, while a modest but statistically significant increase was observed at 100 µM under control conditions (Figure 8A). Two-way ANOVA revealed significant main effects of LPS exposure and compound dose, as well as a significant LPS × dose interaction (LPS: *F* = 5791.780, *p* < 0.001; dose: *F* = 7.664, *p* < 0.001; interaction: *F* = 38.754, *p* < 0.001). Ethyl gallate also induced a modest increase in *Nos2* expression at 100 µM in control cells, but caused a clear dose-dependent reduction in *Nos2* expression in LPS-treated macrophages across all tested concentrations (Figure 8B). The Kruskal–Wallis test revealed significant differences among doses (*H* = 67.031, *p* ≤ 0.001). In contrast, epicatechin produced a minor increase in *Nos2* expression at 100 µM under control conditions and significantly decreased *Nos2* expression only at the highest concentration in LPS-exposed cells (Figure 8C). The Kruskal–Wallis test also revealed significant differences among doses (*H* = 62.139, *p* ≤ 0.001). These results indicate that ethyl gallate exerts the most pronounced and dose-dependent inhibitory effect on LPS-induced *Nos2* expression, whereas ellagic acid suppresses *Nos2* across all tested concentrations without a clear dose dependence, and epicatechin exhibits a weaker inhibitory effect limited to high concentrations.

### 3.8. Effects of Major Phenolic Compounds of RBE on LPS-Induced Ptgs2 and Tnfa mRNA Expression in Macrophages

**Figure 9:**
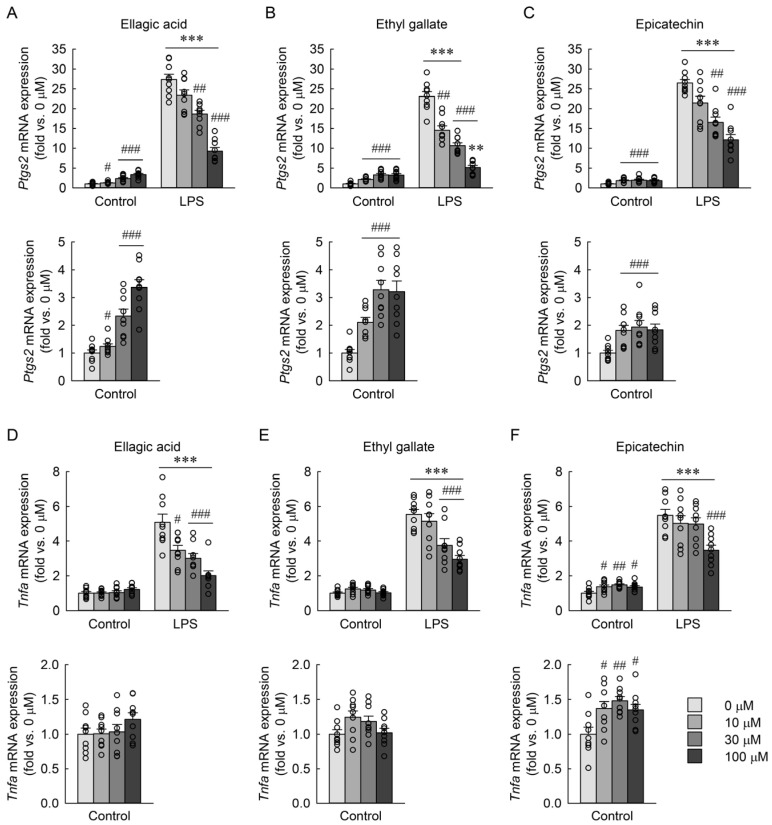
Effects of phytochemical compounds on mRNA expression of *Ptgs2* and *Tnfa* in macrophages exposed to LPS. RAW 264.7 macrophages were treated with ellagic acid, ethyl gallate, and epicatechin at concentrations of 0, 10, 30, and 100 µM for 3 h, followed by exposure to 1 µg/mL LPS for 18 h (*n* = 9). *Ptgs2* mRNA expression of ellagic acid (**A**), ethyl gallate (**B**), and epicatechin (**C**). *Tnfa* mRNA expression of ellagic acid (**D**), ethyl gallate (**E**), and epicatechin (**F**). The control group with lower values is additionally shown in the graph below to improve visualization due to scale differences. Statistical significance was determined by two-way ANOVA followed by Holm–Sidak’s post hoc test. ** *p* < 0.01, *** *p* < 0.001 versus control; # *p* < 0.05, ## *p* < 0.01, ### *p* < 0.001 versus 0 µM.

As shown in Figure 9A, ellagic acid slightly and dose-dependently increased *Ptgs2* mRNA expression in the control group, but significantly reduced expression at 30 and 100 µM following LPS exposure, without a clear dose–response relationship. Two-way ANOVA revealed significant main effects of LPS exposure (*F* = 1507.599, *p* < 0.001) and compound dose (*F* = 2.852, *p* = 0.044), as well as a significant LPS × dose interaction (*F* = 72.829, *p* < 0.001). Ethyl gallate weakly but significantly upregulated *Ptgs2* expression at all concentrations in control cells, while producing a pronounced and concentration-related suppression in LPS-exposed macrophages (Figure 9B). Two-way ANOVA revealed significant effects of LPS (*F* = 596.071, *p* < 0.001), dose (*F* = 6.590, *p* < 0.001), and their interaction (*F* = 69.017, *p* < 0.001). Epicatechin similarly increased *Ptgs2* expression at all concentrations in control cells, although without a clear dose dependence, and reduced expression at 30 and 100 µM under LPS exposure (Figure 9C). Two-way ANOVA revealed significant effects of LPS (*F* = 1413.571, *p* < 0.001), dose (*F* = 3.546, *p* = 0.019), and their interaction (*F* = 21.577, *p* < 0.001). For *Tnfa* mRNA expression (Figure 9D–F), ellagic acid had no effect in control cells but significantly reduced expression at all concentrations in LPS-exposed macrophages, without a clear dose–response relationship (Figure 9D). Two-way ANOVA revealed significant effects of LPS (*F* = 281.684, *p* < 0.001), dose (*F* = 5.824, *p* = 0.001), and their interaction (*F* = 13.338, *p* < 0.001). Ethyl gallate showed a similar pattern, suppressing *Tnfa* at 30 and 100 µM in LPS-treated macrophages (Figure 9E). Two-way ANOVA revealed significant effects of LPS (*F* = 634.514, *p* < 0.001), dose (*F* = 9.534, *p* < 0.001), and their interaction (*F* = 8.010, *p* < 0.001). In contrast, epicatechin slightly increased *Tnfa* expression at all concentrations in the control cells and significantly reduced expression only at 100 µM under LPS exposure (Figure 9F). Two-way ANOVA revealed significant effects of LPS (*F* = 547.829, *p* < 0.001), dose (*F* = 3.688, *p* = 0.016), and their interaction (*F* = 9.159, *p* < 0.001). These results indicate that ellagic acid and ethyl gallate effectively suppress LPS-induced *Ptgs2* and *Tnfa* expression, with ethyl gallate exhibiting a more pronounced concentration-related inhibitory profile, whereas epicatechin displays only partial inhibition at high concentrations.

### 3.9. Effects of Major Phenolic Compounds of RBE on LPS-Induced Il1b and Il6 mRNA Expression in Macrophages

**Figure 10:**
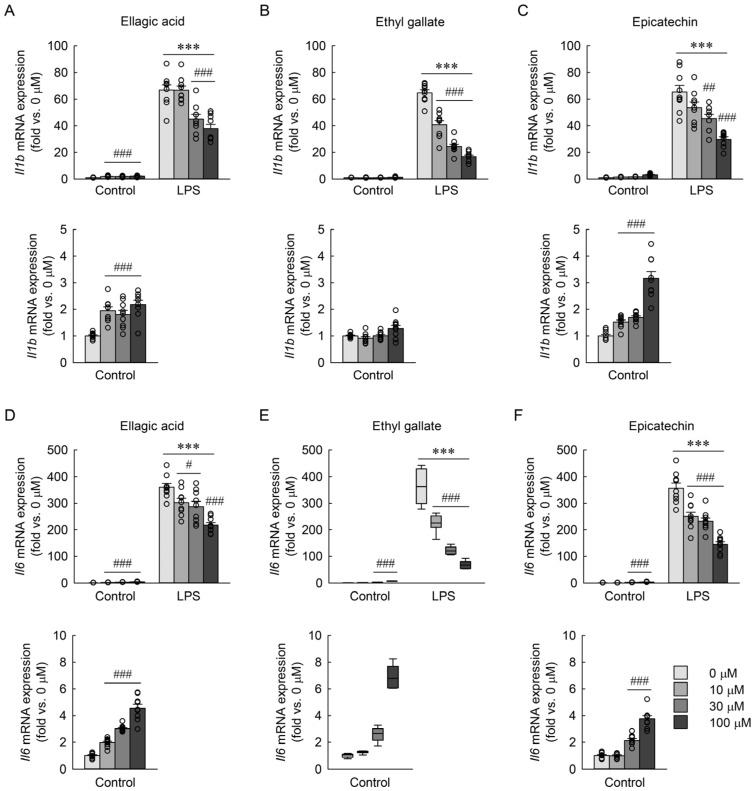
Effects of phytochemical compounds on mRNA expression of *Il1b* and *Il6* in macrophages exposed to LPS. RAW 264.7 macrophages were treated with ellagic acid, ethyl gallate, and epicatechin at concentrations of 0, 10, 30, and 100 µM for 3 h, followed by exposure to 1 µg/mL LPS for 18 h (*n* = 9). *Il1b* mRNA expression of ellagic acid (**A**), ethyl gallate (**B**), and epicatechin (**C**). *Il6* mRNA expression of ellagic acid (**D**), ethyl gallate (**E**), and epicatechin (**F**). The control group with lower values is additionally shown in the graph below to improve visualization due to scale differences. Statistical significance was determined using two-way ANOVA followed by Holm–Sidak’s post hoc test for ellagic acid (**A**,**D**), ethyl gallate (**B**,**E**), and epicatechin (**C**,**F**), and a two-factor linear model with HC3 standard errors followed by Holm-corrected pairwise comparisons for *Il6* expression following ethyl gallate treatment (**E**). *** *p* < 0.001 versus control; # *p* < 0.05, ## *p* < 0.01, ### *p* < 0.001 versus 0 µM.

For *Il1b* mRNA expression, ellagic acid weakly increased expression in control cells at all concentrations, but significantly decreased *Il1b* levels at 30 and 100 µM in the LPS group, without dose dependency (Figure 10A). Two-way ANOVA revealed significant effects of LPS (*F* = 4319.660, *p* < 0.001), dose (*F* = 7.336, *p* < 0.001), and their interaction (*F* = 28.929, *p* < 0.001). Ethyl gallate had no effect in control cells but strongly and dose-dependently inhibited *Il1b* expression at all concentrations under LPS stimulation (Figure 10B). Two-way ANOVA revealed significant effects of LPS (*F* = 5084.276, *p* < 0.001), dose (*F* = 28.744, *p* < 0.001), and their interaction (*F* = 52.276, *p* < 0.001). Epicatechin tended to increase *Il1b* mRNA expression in control cells across concentrations, although this effect was not dose-dependent, and significantly suppressed *Il1b* expression at 30 and 100 µM in the LPS-treated group (Figure 10C). Two-way ANOVA revealed significant effects of LPS (*F* = 4728.432, *p* < 0.001) and their interaction (*F* = 69.053, *p* < 0.001), but not dose (*F* = 2.397, *p* = 0.076). For *Il6* mRNA expression, both ellagic acid and ethyl gallate induced modest but significant, dose-dependent increases in the control cells and reduced expression in LPS-treated cells at all concentrations (Figure 10D,E), though the inhibitory effect of ellagic acid was not dose-dependent (Figure 10D). Two-way ANOVA for ellagic acid revealed significant effects of LPS (*F* = 15,200.554, *p* < 0.001), dose (*F* = 32.994, *p* < 0.001), and their interaction (*F* = 117.185, *p* < 0.001). Ethyl gallate exhibited a clear dose-dependent decrease in *Il6* expression under LPS stimulation (Figure 10E). A two-factor linear model with HC3 standard errors revealed significant main effects of LPS (*F* = 5507.88, *p* < 0.001), dose (*F* = 428.33, *p* < 0.001), and their interaction (*F* = 393.23, *p* < 0.001). Epicatechin slightly increased *Il6* expression at 30 and 100 µM under control conditions but reduced it at all concentrations following LPS exposure, without a clear dose-dependent trend (Figure 10F). Two-way ANOVA revealed significant effects of LPS (*F* = 11,719.022, *p* < 0.001), dose (*F* = 15.206, *p* < 0.001), and their interaction (*F* = 118.786, *p* < 0.001). These results indicate that ellagic acid and ethyl gallate effectively suppress LPS-induced *Il1b* and *Il6* expression, whereas epicatechin exhibits weaker and less consistent inhibitory effects.

### 3.10. Correlation Between NO Production and Pro-Inflammatory mRNA Expression in Macrophages

**Figure 11:**
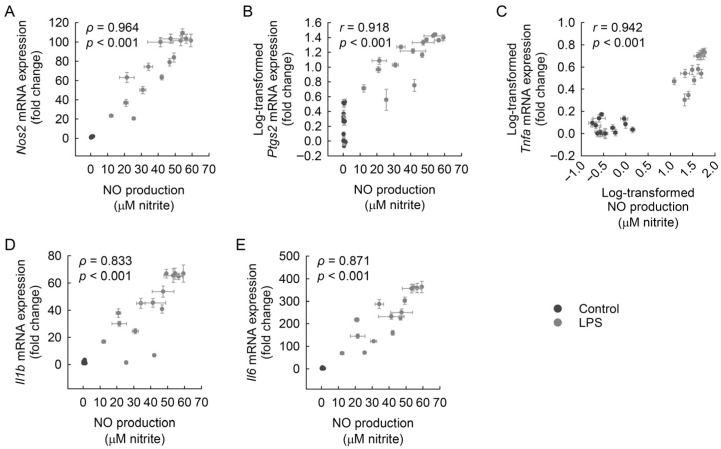
Correlation between NO production and mRNA expression levels of *Nos2*, *Ptgs2*, *Tnfa*, *Il1b*, and *Il6* in macrophages exposed to LPS. RAW 264.7 macrophages were pretreated with RBE (0, 50, and 100 µg/mL) or phytochemical compounds—ellagic acid, ethyl gallate, and epicatechin (0, 10, 30, and 100 µM)—for 3 h, followed by exposure to 1 µg/mL LPS for 18 h. Correlations between NO production and mRNA expression were analyzed in both control (unexposed to LPS) and LPS-treated groups. Relationships between NO production and mRNA expressions of *Nos2* (**A**), *Ptgs2* (**B**), *Tnfa* (**C**), *Il1b* (**D**), and *Il6* (**E**) were examined using pooled data from all treatment conditions (*n* = 30). Statistical correlations were determined using Spearman’s rank correlation for non-parametric associations (**A**,**D**,**E**) and Pearson’s correlation for linear associations (**B**,**C**), yielding Spearman’s *ρ* and Pearson’s *r*, respectively.

To further explore the link between NO production and inflammatory gene activation, correlations were analyzed between NO levels and the mRNA expression of *Nos2*, *Ptgs2*, *Tnfa*, *Il1b*, and *Il6*. As shown in Figure 11A–E, strong positive correlations were observed for all five genes in LPS-exposed macrophages. *Nos2* showed the highest correlation (*ρ* = 0.964, *p* < 0.001), followed by *Ptgs2* (*r* = 0.918, *p* < 0.001), *Tnfa* (*r* = 0.942, *p* < 0.001), *Il1b* (*ρ* = 0.833, *p* < 0.001), and *Il6* (*ρ* = 0.871, *p* < 0.001). These results indicate that NO production is closely associated with the transcriptional activation of key inflammatory genes in LPS-stimulated macrophages.

### 3.11. Effects of Short-Term NO Induction and Exogenous NO Donors on Macrophage Responses

**Figure 12:**
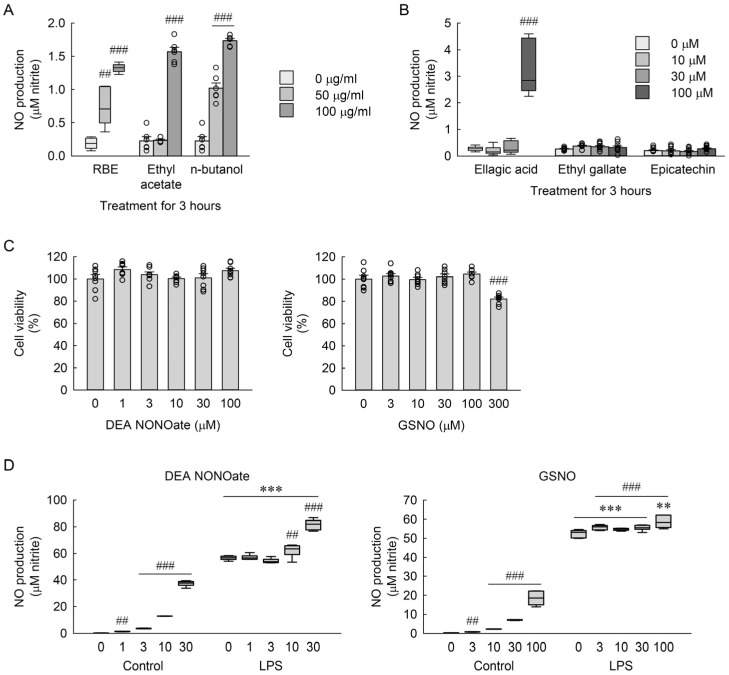
Effects of RBE, its fractions, purified compounds, and NO donors on NO production and cell viability in macrophages. (**A**) NO production in RAW 264.7 macrophages treated with RBE, ethyl acetate fraction, or n-butanol fraction at concentrations of 0, 50, and 100 µg/mL for 3 h (*n* = 6 for RBE and fractions, and *n* = 9 for compounds). Statistical significance was determined using Welch’s one-way ANOVA followed by Holm-corrected Welch’s *t*-test for RBE, and one-way ANOVA followed by Holm–Sidak’s post hoc test for ethyl acetate and n-butanol fractions. (**B**) NO production in RAW 264.7 macrophages treated with ellagic acid, ethyl gallate, and epicatechin at concentrations of 0, 10, 30, and 100 µM for 3 h. Statistical significance was determined using Welch’s one-way ANOVA followed by Holm-corrected Welch’s *t*-test for ellagic acid, and two-way ANOVA followed by Holm–Sidak’s post hoc test for ethyl gallate and epicatechin. (**C**) Cell viability in RAW 264.7 macrophages treated with diethylamine NONOate diethylammonium salt (DEA NONOate; 0, 1, 3, 10, 30, and 100 µM) or S-nitrosoglutathione (GSNO; 0, 3, 10, 30, 100, and 300 µM) for 27 h (*n* = 8). Statistical significance was determined using one-way ANOVA followed by Holm–Sidak’s post hoc test. (**D**) NO production in RAW 264.7 macrophages treated with DEA NONOate (0, 1, 3, 10, and 30 µM) and GSNO (0, 3, 10, 30, 100, and 300 µM) for 3 h, followed by exposure to 1 µg/mL LPS for 24 h (*n* = 6). Statistical significance was determined using the Kruskal–Wallis test followed by Dunn’s post hoc test. ** *p* < 0.01, *** *p* < 0.001 versus control; ## *p* < 0.01, ### *p* < 0.001 versus 0 µg/mL or 0 µM.

To assess whether the slight increase in NO observed after 3 h of RBE or phenolic compound treatment is associated with subsequent inflammatory responses, a short-term preconditioning experiment was performed. As shown in Figure 12A,B, brief exposure (3 h) to RBE, its active fractions, or major compounds induced a mild but statistically significant increase in NO production under basal conditions. The n-butanol and ethyl acetate fractions produced the greatest increases, whereas individual compounds elicited smaller changes, most notably at 100 µM. For RBE and ellagic acid, Welch’s one-way ANOVA revealed significant differences among treatment groups (*F* = 57.4, *p* < 0.001 for RBE; *F* = 41.54, *p* < 0.001 for ellagic acid). One-way ANOVA indicated significant group effects for the ethyl acetate and n-butanol fractions (*F* = 206.183, *p* < 0.001 and *F* = 149.781, *p* < 0.001, respectively), but not for ethyl gallate or epicatechin (*F* = 1.236, *p* = 0.313 and *F* = 1.447, *p* = 0.248 for epicatechin, respectively). Pretreatment with exogenous NO donors (DEA NONOate and GSNO) showed that neither compound was cytotoxic at concentrations up to 100 or 300 µM (Figure 12C). One-way ANOVA revealed significant group differences for GSNO (*F* = 15.736, *p* < 0.001), but not for DEA NONOate (*F* = 2.241, *p* = 0.068). However, NO donor preconditioning did not suppress LPS-induced NO production; instead, it further enhanced NO levels in a concentration-dependent manner (Figure 12D). Consistently, the Kruskal–Wallis test indicated significant differences among treatment groups for both DEA NONOate and GSNO (*H* = 56.593, *p* ≤ 0.001 and *H* = 55.439, *p* ≤ 0.001 for GSNO, respectively). These results are consistent with the notion that the transient NO increase induced by RBE and its fractions reflects short-term activation rather than a protective preconditioning effect prior to subsequent LPS stimulation.

## 4. Discussion

In this study, RBE and its phenolic-enriched fractions exhibited antioxidant activity and attenuated inflammatory responses in LPS-stimulated RAW 264.7 macrophages. RBE and its phenolic-enriched solvent fractions significantly suppressed LPS-induced NO production without affecting cell viability and concomitantly reduced the mRNA expression of major pro-inflammatory mediators, including *Nos2*, *Ptgs2*, *Tnfa*, *Il1b*, and *Il6*. In addition, three major phenolic constituents—ellagic acid, ethyl gallate, and epicatechin—were identified in the active fractions and were shown to individually attenuate NO production and inflammatory gene expression, albeit with distinct response patterns. Together, these findings suggest that phenolic constituents contribute to the attenuation of NO-associated inflammatory responses in macrophages.

A central observation of this study is that the ethyl acetate and n-butanol fractions exhibited the strongest antioxidant capacity and the most pronounced inhibition of LPS-induced NO production. These fractions showed the highest total phenolic content and superior radical-scavenging and reducing activities across DPPH, ABTS, and FRAP assays. Given the close relationship between oxidative stress and macrophage activation, the enrichment of redox-active phenolic compounds in these fractions likely contributes to their anti-inflammatory effects [35,36]. This fractionation-based approach supports the interpretation that phenolic-rich fractions make a substantial contribution to the observed bioactivity of RBE [37,38]. Consistent with this fractionation-dependent enrichment of phenolic bioactivity, RBE coordinately suppressed the transcriptional induction of multiple inflammatory mediators central to LPS-driven macrophage activation. While previous studies on *R*. *buergeri* leaf extracts reported reductions in NO and prostaglandin production at the protein level [30], the present work extends these observations by demonstrating coordinated inhibition at the mRNA level across *Nos2*, *Ptgs2*, and key pro-inflammatory cytokines. The coordinated reduction in these transcripts suggests that RBE affects multiple components of the LPS-responsive inflammatory program rather than a single downstream mediator [28,39]. Although intracellular signaling pathways were not directly examined, the transcriptional profile observed here is consistent with suppression of canonical LPS-responsive cascades, including NF-κB and MAPK pathways [40,41]. To strengthen the biological relevance of these transcriptional targets, we integrated our experimental findings with bioinformatics analysis of an independent LPS-stimulated macrophage gene expression dataset. Enrichment of inflammatory GO terms and identification of hub genes within the “cellular response to lipopolysaccharide” network highlighted *Nos2*, *Ptgs2*, *Tnfa*, and *Il1b* as central nodes in macrophage inflammatory responses [42,43]. The overlap between bioinformatically identified hub genes and experimentally examined targets supports the biological relevance of the genes selected in this study.

Chemical profiling suggested ellagic acid, ethyl gallate, and epicatechin as major phenolic constituents of the active fractions. Functional evaluation further demonstrated that these compounds contribute differentially to the anti-inflammatory activity of RBE, with ethyl gallate exerting the most consistent and dose-dependent suppression of LPS-induced *Nos2* and cytokine expression, whereas ellagic acid reduced inflammatory markers across concentrations without a clear monotonic dose–response relationship. Epicatechin displayed comparatively weaker inhibitory effects, with significant suppression observed primarily at higher concentrations for several endpoints. These compound-specific response patterns suggest that the anti-inflammatory activity of RBE likely arises from additive or complementary actions of multiple phenolic constituents rather than from a single dominant compound [44,45]. Because the present chemical analysis focused on major UV-detectable phenolics, the contribution of additional minor constituents cannot be excluded. At the system level, the functional linkage between NO production and inflammatory gene activation was further reinforced by correlation analyses, which revealed strong positive associations between NO levels and the expression of *Nos2*, *Ptgs2*, *Tnfa*, *Il1b*, and *Il6* in LPS-exposed macrophages [46,47]. The particularly strong correlation with *Nos2* is consistent with the role of iNOS as the primary source of NO under inflammatory conditions, while robust correlations with cytokine transcripts indicate that NO production closely reflects the overall inflammatory transcriptional state [48,49]. These findings support the use of NO as an integrative marker of macrophage inflammatory activation in this experimental context.

Interestingly, short-term exposure to RBE, its active fractions, or individual phenolic compounds induced a modest increase in basal NO production under non-stimulated conditions. However, preconditioning macrophages with exogenous NO donors failed to attenuate subsequent LPS-induced NO production and instead further amplified it in a concentration-dependent manner. These findings suggest that the transient increase in basal NO does not confer tolerance to subsequent LPS stimulation and is more consistent with an early cellular response than with a protective preconditioning effect [50,51]. This context-dependent behavior underscores that NO modulation by RBE is selective for pathological, LPS-driven inflammatory activation rather than indiscriminate suppression or induction of NO signaling.

Several limitations of this study should be acknowledged. First, all experiments were conducted in a single macrophage cell line, and validation in primary macrophages and in vivo models will be required to establish physiological relevance. Second, inflammatory regulation was primarily assessed at the transcriptional level; future studies should incorporate protein-level measurements and direct evaluation of upstream signaling pathways. Third, although major phenolic constituents were tentatively identified based on retention time and UV spectra, MS fragmentation analysis was performed to support compound identification; however, full structural confirmation using high-resolution MS or nuclear magnetic resonance (NMR) was not conducted, and comprehensive metabolomic profiling will be necessary to fully define the bioactive composition of RBE and to evaluate potential synergistic interactions among its components.

## 5. Conclusions

This study demonstrates that *R*. *buergeri* extract and phenolic-enriched solvent fractions possess strong antioxidant and anti-inflammatory activities in macrophages. RBE suppresses LPS-induced NO production and coordinately downregulates key inflammatory genes central to macrophage activation. The tentative identification and functional testing of ellagic acid, ethyl gallate, and epicatechin provide compound-level support for the contribution of phenolics to the bioactivity of RBE. Collectively, these findings suggest that *R*. *buergeri* represents a promising source of bioactive phenolic compounds, warranting further investigation in in vivo models.

## Figures and Tables

**Table 1:** Primer sequences used for qRT-PCR analysis.

Gene	Forward Primer (5′–3′)	Reverse Primer (5′–3′)
*Gapdh*	ATC ACT GCC ACC CAG AA	TCC ACG ACG GAC ACA TTG
*Nos2*	CAG CTG GGC TGT ACA AAC CTT	CAT TGG AAG TGA AGC GTT TCG
*Ptgs2*	TGA GTG GGG TGA TGA GCA AC	AAG TGG TAA CCG CTC AGG TG
*Tnfa*	TCT TCT CAT TCC TGC TTG TGG	GAG GCC ATT TGG GAA CTT CT
*Il1b*	GCC ACC TTT TGA CAG TGA TGA G	GAC AGC CCA GGT CAA AGG TT
*Il6*	AGT TGC CTT CTT GGG ACT GA	TCC ACG ATT TCC CAG AGA AC

**Table 2:** IC_50_ values of RBE and its fractions for DPPH free radical scavenging activity.

Sample	IC_50_ (μg/mL)	*p*
*Rubus buergeri* extract	35.3 (34.7–35.4)	††,‡
Hexane lower layer	29.2 (29.2–29.4)	†
Hexane upper layer	862.7 (852.7–864.9)	††,‡‡
Methyl chloride	179.0 (177.7–179.9)	††,‡‡
Ethyl acetate	8.8 (8.7–8.9)	
n-Butanol	12.9 (12.6–13.3)	
Hydrated butanol	32.7 (32.2–33.0)	†,‡
Water	267.0 (266.8–267.5)	††,‡‡

DPPH free radical scavenging activity was evaluated for RBE and its fractions. The results are expressed as IC_50_ values, presented as median (IQR) from three independent measurements (*n* = 3). The Kruskal–Wallis test indicated a significant difference in the IC_50_ values of RBE and its fractions in scavenging DPPH free radicals (*H* = 22.680 with 7 degrees of freedom, *p* = 0.002). Post hoc multiple comparisons were performed for all pairwise group comparisons using Dunn’s test. Because the ethyl acetate and n-butanol fractions exhibited the lowest IC_50_ values, comparisons relative to these two fractions are specifically indicated in the table. Additionally, significant differences were observed with † *p* < 0.05, †† *p* < 0.01 versus the ethyl acetate fraction; ‡ *p* < 0.05, ‡‡ *p* < 0.01 versus the n-butanol fraction. Butylated hydroxytoluene (BHT) and L-ascorbic acid (LAA) were used as positive controls in the DPPH assay, with IC_50_ values of 57.2 ± 13.8 µg/mL and 5.2 ± 1.9 µg/mL (mean ± SD), respectively.

**Table 3:** Antioxidant properties of RBE and its fractions assessed by the ABTS assay.

Sample	TEAC (mM/g)	*p*
*Rubus buergeri* extract	2.23 ± 0.02	††,‡
Hexane lower layer	1.32 ± 0.01	†
Hexane upper layer	3.15 ± 0.03	††,‡‡
Methyl chloride	1.95 ± 0.01	††,‡‡
Ethyl acetate	5.03 ± 0.06	
n-Butanol	4.37 ± 0.02	
Hydrated butanol	2.83 ± 0.04	†,‡
Water	0.89 ± 0.05	††,‡‡

The antioxidant properties of RBE and its fractions were evaluated using the ABTS assay. The results are expressed as TEAC (mM/g dry weight) and are presented as mean ± standard error of the mean (SEM) (*n* = 6). Welch’s one-way ANOVA indicated a significant difference in the TEAC values among RBE and its fractions (*F* = 2736.23, *p* < 0.001). Additionally, significant differences were observed with † *p* < 0.05, †† *p* < 0.01 versus the ethyl acetate fraction; ‡ *p* < 0.05, ‡‡ *p* < 0.01 versus the n-butanol fraction, as determined by post hoc multiple comparisons using the Games-Howell test. Trolox was used as the reference standard for calculation of TEAC values.

**Table 4:** Antioxidant properties of RBE and its fractions assessed by the FRAP assay.

Sample	TEAC (mM/g)	*p*
*Rubus buergeri* extract	263.4 (256.7–272.4)	†††,‡‡‡
Hexane lower layer	295.7 (288.4–439.9)	†††,‡‡‡
Hexane upper layer	49.1 (47.3–50.8)	†††,‡‡‡
Methyl chloride	190.3 (189.3–190.7)	†††,‡‡‡
Ethyl acetate	969.0 (963.1–978.0)	‡‡
n-Butanol	792.6 (781.3–802.5)	††
Hydrated butanol	427.4 (425.4–428.3)	†††,‡‡‡
Water	80.1 (78.8–84.0)	†††,‡‡‡

The antioxidant properties of RBE and its fractions were evaluated using the FRAP assay. The results are expressed as TEAC (mM/g dry weight), presented as the median (IQR) (*n* = 7). The Kruskal–Wallis test indicated a significant difference in TEAC values among RBE and its fractions (*H* = 46.288 with 7 degrees of freedom, *p* ≤ 0.001). Post hoc multiple comparisons were performed using Dunn’s test. Comparisons relative to the ethyl acetate and n-butanol fractions are indicated in the table, with †† *p* < 0.01, ††† *p* < 0.001 versus the ethyl acetate fraction; ‡‡ *p* < 0.01 and ‡‡‡ *p* < 0.001 versus the n-butanol fraction. Trolox was used as the reference standard for calculation of TEAC values.

**Table 5:** Topological parameters of genes associated with the GO biological process “cellular response to lipopolysaccharide” in the Search Tool for the Retrieval of Interacting Genes (STRING) PPI network.

Gene Symbol	Degree	Betweenness Centrality	Closeness Centrality	Stress	Neighborhood Connectivity
*Il1b*	37	0.069	0.833	1280	21.2
*Tnfa*	36	0.062	0.818	1184	21.2
*Stat1*	34	0.119	0.789	1562	21.5
*Il6*	33	0.033	0.776	782	22.4
*Cxcl10*	33	0.056	0.776	1098	22.3
*Casp1*	31	0.022	0.738	462	23.2
*Ccl2*	31	0.025	0.750	576	23.0
*Myd88*	29	0.019	0.714	434	23.7
*Nfkb1*	28	0.025	0.703	454	22.6
*Ptgs2*	28	0.012	0.703	302	24.0
*Il1a*	28	0.011	0.703	300	24.0
*Il18*	27	0.012	0.692	294	24.3
*Cxcl9*	26	0.025	0.692	600	23.6
*Cd274*	25	0.022	0.682	606	24.6
*Nos2*	24	0.004	0.662	104	25.6
*Tnfaip3*	24	0.023	0.672	454	22.6
*Csf3*	24	0.009	0.672	270	25.4
*Cd80*	22	0.003	0.643	70	26.4
*Jak2*	21	0.001	0.634	40	27.4
*Il12b*	20	0.006	0.634	160	26.9
*Cxcl2*	20	0.002	0.608	74	26.5
*Nlrp3*	19	0.004	0.616	116	26.8
*Src*	18	0.003	0.608	72	26.1
*Cebpb*	18	0.006	0.616	170	25.8
*Gbp2b*	17	0.016	0.608	454	22.5
*Irgm1*	17	0.010	0.608	338	23.2
*Nod2*	17	0.002	0.600	74	27.1
*Gbp2*	16	0.007	0.600	270	23.4
*Nfkbiz*	16	0.005	0.556	68	23.6
*Nfkbib*	14	0.003	0.577	66	27.4
*Gbp5*	14	0.009	0.577	210	21.1
*Serpine1*	13	0.000	0.556	6	28.5
*Zc3h12a*	12	0.046	0.529	780	21.3
*Gbp3*	12	0.002	0.563	96	22.5
*Irgm2*	12	0.022	0.523	780	18.2
*Igtp*	12	0.022	0.523	780	18.2
*Ripk2*	11	0.000	0.517	2	28.1
*Tnfrsf1b*	11	0.000	0.542	6	29.6
*Cmpk2*	10	0.002	0.511	24	17.2
*Zfp36*	9	0.001	0.511	10	25.4
*Malt1*	6	0.000	0.495	4	25.5
*Epsti1*	5	0.002	0.464	6	15.4
*Tnip1*	5	0.000	0.474	0	26.2
*Plscr1*	2	0.000	0.450	0	19.5
*Gbp10*	2	0.000	0.349	0	12.0
*Arid5a*	1	0.000	0.349	0	12.0

Upregulated genes (log_2_ fold change > 1 and FDR-adjusted *p* < 0.05) identified from the GSE76562 dataset were subjected to GO enrichment analysis using DAVID. The 49 genes classified under the GO biological process “cellular response to lipopolysaccharide” were further analyzed using STRING to construct the PPI network. Network topology parameters—including degree, betweenness centrality, closeness centrality, stress, and neighborhood connectivity—were calculated in STRING to assess the relative importance of each gene within the LPS-responsive network.

## Data Availability

The original data presented in this study are included in the article and Supplementary File S1.

## Supplementary Materials

The following supporting information can be downloaded at: https://www.mdpi.com/article/10.3390/cimb48050507/s1.

## Abbreviations

The following abbreviations are used in this manuscript:

ABTS	2,2′-azino-bis(3-ethylbenzothiazoline-6-sulfonic acid)
ANOVA	analysis of variance
BHT	butylated hydroxytoluene
cDNA	complementary DNA
DMSO	dimethyl sulfoxide
DMEM	Dulbecco’s Modified Eagle Medium
DPPH	2,2-diphenyl-1-picrylhydrazyl
FRAP	ferric reducing antioxidant power
GAE	gallic acid equivalents
GEO	Gene Expression Omnibus
GO	Gene Ontology
HC3	heteroscedasticity-consistent standard errors (type 3)
IC_50_	half-maximal inhibitory concentration
iNOS	inducible nitric oxide synthase
LPS	lipopolysaccharide
MCC	maximal clique centrality
MTT	3-(4,5-dimethylthiazol-2-yl)-2,5 diphenyltetrazolium bromide
NO	nitric oxide
qRT-PCR	quantitative reverse transcription polymerase chain reaction
RBE	*Rubus buergeri* extract
ROS	reactive oxygen species
SEM	standard error of the mean
STRING	Search Tool for the Retrieval of Interacting Genes
TFC	total flavonoid content
TEAC	Trolox equivalent antioxidant capacity
TPC	total phenolic content
