# Supplementary material for: Phenolic-Enriched Fractions of Rubus buergeri Attenuate LPS-Induced Nitric Oxide Production and Inflammatory Gene Expression in Macrophages
Source: Curr Issues Mol Biol. 2026 May 14;48(5):507. doi: 10.3390/cimb48050507 (PMC13204866; doi:10.3390/cimb48050507)
Supplement: Supplementary file 1 [file cimb-48-00507-s001.zip › cimb-4276456-supplementary.pdf]

Supplementary File S1: Raw data and statistical results.

DPPH RADICAL SCAVENGING ACTIVITY

IC_{50} (µg/ml)

Experiment	RBE	L-Hexane	U-Hexane	MC	EA	n-BuOH	H BuOH	Water
#1	34.0	842.8	29.6	179.0	8.6	13.6	31.6	267.0
#2	35.3	862.7	29.2	180.7	9.0	12.9	32.7	267.9
#3	35.5	867.1	29.2	176.3	8.8	12.4	33.3	266.6
mean	34.9	857.5	29.4	178.7	8.8	13.0	32.5	267.2
SD	0.8	12.9	0.2	2.2	0.2	0.6	0.8	0.7
SEM	0.5	6.5	0.1	1.3	0.1	0.3	0.4	0.4
**median**	**35.3**	**862.7**	**29.2**	**179.0**	**8.8**	**12.9**	**32.7**	**267.0**
**Q1**	**34.7**	**852.7**	**29.2**	**177.7**	**8.7**	**12.6**	**32.2**	**266.8**
**Q3**	**35.4**	**864.9**	**29.4**	**179.9**	**8.9**	**13.3**	**33.0**	**267.5**
vs. EA	0.009	0.007	0.020	0.008		0.050	0.012	0.007
vs. P-BuOH	0.012	0.007	0.050	0.009	0.050		0.020	0.008

Normality Test (Shapiro-Wilk): Failed ($p < 0.050$)

Kruskal-Wallis Test: H = 22.680 with 7 degrees of freedom ($p = 0.002$)

ABTS RADICAL SCAVENGING ACTIVITY

TEAC, Trolox equivalent antioxidant concentration (mM/g dry weight)

Experiment	RBE	Hexane L	Hexane U	MC	EA	n-BuOH	H BuOH	Water
#1	2.27	1.35	3.06	1.92	5.18	4.41	2.72	0.99
#2	2.28	1.35	3.08	1.95	5.17	4.42	2.75	1.01
#3	2.28	1.29	3.08	1.97	5.16	4.41	2.77	1.01
#4	2.20	1.33	3.23	1.94	4.90	4.34	2.89	0.78
#5	2.18	1.31	3.23	1.96	4.88	4.32	2.92	0.78
#6	2.16	1.29	3.22	1.95	4.88	4.33	2.92	0.80
**mean**	**2.23**	**1.32**	**3.15**	**1.95**	**5.03**	**4.37**	**2.83**	**0.89**
**SD**	**0.05**	**0.03**	**0.08**	**0.02**	**0.16**	**0.05**	**0.09**	**0.12**
**SEM**	**0.02**	**0.01**	**0.03**	**0.01**	**0.06**	**0.02**	**0.04**	**0.05**
vs. EA	<0.001	<0.001	<0.001	<0.001		<0.001	<0.001	<0.001
vs. P-BuOH	<0.001	<0.001	<0.001	<0.001	0.005		<0.001	<0.001

Normality Test (Shapiro-Wilk): Passed ($p = 0.449$)

Equal Variance Test (Brown-Forsythe): Failed ($p < 0.050$)

Welch's One-Way ANOVA: F(7, 16.43) = 2736.23 ($p < 0.001$)

FRAP

TEAC, Trolox equivalent antioxidant concentration (mM/g dry weight)

Experiment	RBE	Hexane L	Hexane U	MC	EA	n-BuOH	H BuOH	Water
#1	265.2	49.1	364.4	190.0	969.0	792.6	427.7	81.5
#2	280.3	52.8	295.7	190.9	985.3	807.9	427.4	77.5
#3	263.4	49.1	515.4	190.9	966.0	783.2	424.5	80.1
#4	250.5	45.5	281.1	188.6	972.5	801.7	428.8	86.7
#5	279.6	52.5	295.7	190.5	983.6	777.0	426.2	77.1
#6	262.8	49.1	515.6	190.3	947.6	779.4	424.5	80.0
#7	250.0	45.4	280.7	187.8	960.3	803.2	430.4	86.5
mean	264.5	49.1	364.1	189.8	969.2	792.1	427.1	81.4
SD	12.2	3.0	107.3	1.2	13.1	12.5	2.2	3.9
SEM	4.6	1.1	40.5	0.5	5.0	4.7	0.8	1.5
**median**	**263.4**	**49.1**	**295.7**	**190.3**	**969.0**	**792.6**	**427.4**	**80.1**
**Q1**	**256.7**	**47.3**	**288.4**	**189.3**	**963.1**	**781.3**	**425.4**	**78.8**
**Q3**	**272.4**	**50.8**	**439.9**	**190.7**	**978.0**	**802.5**	**428.3**	**84.0**
vs. EA	<0.001	<0.001	<0.001	<0.001		0.002	<0.001	<0.001
vs. P-BuOH	<0.001	<0.001	<0.001	<0.001	0.002		<0.001	<0.001

Normality Test (Shapiro-Wilk): Failed ($p < 0.050$)

Kruskal-Wallis Test: H = 53.633 with 7 degrees of freedom (p =< 0.001)

TOTAL PHENOLIC CONTENT

mg gallic acid equivalent/g DW dry weight

Experiment	RBE	Hexane L	Hexane U	MC	EA	n-BuOH	H BuOH	Water
#1	101.1	13.4	127.4	102.0	331.1	225.6	127.7	22.2
#2	103.2	20.9	132.8	103.6	433.8	267.3	142.1	20.7
#3	109.1	13.6	133.3	14.0	263.5	226.7	146.4	23.9
#4	120.3	17.8	141.7	18.7	329.7	258.5	172.5	25.2
#5	76.0	11.4	86.4	48.6	206.2	180.3	105.2	21.8
#6	88.2	12.3	94.1	56.9	262.8	223.6	152.9	20.9
#7	79.0	11.7	97.7	52.7	217.9	186.7	112.8	21.8
mean	96.7	14.5	116.2	56.7	292.1	224.1	137.1	22.3
SD	16.2	3.6	22.6	35.6	79.1	32.6	23.5	1.6
SEM	6.1	1.3	8.5	13.5	29.9	12.3	8.9	0.6
**median**	**101.1**	**13.4**	**127.4**	**52.7**	**263.5**	**225.6**	**142.1**	**21.8**
**Q1**	**83.6**	**12.0**	**95.9**	**33.7**	**240.3**	**205.2**	**120.3**	**21.3**
**Q3**	**106.1**	**15.7**	**133.0**	**79.5**	**330.4**	**242.6**	**149.7**	**23.1**
vs. EA	<0.001	<0.001	<0.001	<0.001		0.110	<0.001	<0.001
vs. P-BuOH	<0.001	<0.001	<0.001	<0.001	0.110		<0.001	<0.001

Normality Test (Shapiro-Wilk): Failed ($p < 0.050$)

Kruskal-Wallis Test: $H = 50.098$ with 7 degrees of freedom ($p =< 0.001$)

TOTAL FLAVONOID CONTENT

mg quercetin equivalent/g DW dry weight

Experiment	RBE	Hexane L	Hexane U	MC	EA	n-BuOH	H BuOH	Water
#1	23.6		75.2	46.0	19.8		18.0	6.7
#2		124.2						
#3	23.9		72.5	48.1	19.2	21.0	18.0	7.0
#4	18.0	132.0	63.8	41.0	14.3	17.4	13.7	1.3
#5	17.1	128.6	62.3	37.9		16.7	12.3	2.9
#6	17.0	123.4	60.2	38.1	13.7	18.0	13.5	3.6
#7		121.7			12.1	15.7		
mean	19.9	126.0	66.8	42.2	15.8	17.8	15.1	4.3
SD	3.5	4.2	6.7	4.6	3.4	2.0	2.7	2.5
SEM	1.6	1.9	3.0	2.1	1.5	0.9	1.2	1.1
**median**	**18.0**	**124.2**	**63.8**	**41.0**	**14.3**	**17.4**	**13.7**	**3.6**
**Q1**	**17.1**	**123.4**	**62.3**	**38.1**	**13.7**	**16.7**	**13.5**	**2.9**
**Q3**	**23.6**	**128.6**	**72.5**	**46.0**	**19.2**	**18.0**	**18.0**	**6.7**
vs. U-Hexane	<0.001		0.009	0.001	<0.001	<0.001	0.001	<0.001

Normality Test (Shapiro-Wilk): Failed ($p < 0.050$)

Kruskal-Wallis Test: $H = 35.017$ with 7 degrees of freedom ($p =< 0.001$)

CORRELATION vs. TPC

Values

	TPC		DPPH		ABTS		FRAP	
	mean	SEM	mean	SEM	mean	SEM	mean	SEM
**RBE**	96.69	6.14	34.95	0.46	2.23	0.02	264.5	4.6
**Hexane L**	14.45	1.35	857.50	6.47	1.32	0.01	49.1	1.1
**Hexane U**	116.20	8.54	29.37	0.12	3.15	0.03	364.1	40.5
**MC**	56.66	13.45	178.66	1.28	1.95	0.01	189.8	0.5
**EA**	292.14	29.91	8.80	0.09	5.03	0.06	969.2	5.0
**P Butanol**	224.09	12.32	12.96	0.32	4.37	0.02	792.1	4.7
**H Butanol**	137.09	8.88	32.53	0.41	2.83	0.04	427.1	0.8
**Water**	22.35	0.62	267.19	0.39	0.89	0.05	81.4	1.5

Logarithmic transformation

	TPC		DPPH	
	mean	SEM	mean	SEM
**RBE**	1.98	0.03	1.543	0.006
**Hexane L**	1.15	0.04	2.933	0.003
**Hexane U**	2.06	0.03	1.468	0.002
**MC**	1.66	0.13	2.252	0.003
**EA**	2.45	0.04	0.944	0.005
**P Butanol**	2.35	0.02	1.112	0.011
**H Butanol**	2.13	0.03	1.512	0.006
**Water**	1.35	0.01	2.427	0.001

**Normality Test (Shapiro-Wilk):**	Passed (p = 0.805)	Passed (p = 0.649)	Passed (p = 0.979)
**Constant Variance Test:**	Passed (p = 0.460)	Passed (p = 0.705)	Passed (p = 0.885)
**Pearson Correlation:**	r = -0.907	r = 0.981	r = 0.996
	$p < 0.01$	$p < 0.001$	$p < 0.001$

CORRELATION vs. TFC

Values

	TFC		DPPH		ABTS		FRAP	
	mean	SEM	mean	SEM	mean	SEM	mean	SEM
**RBE**	19.9	1.6	34.95	0.46	2.23	0.02	264.5	4.6
**Hexane L**	126.0	1.9	857.50	6.47	1.32	0.01	49.1	1.1
**Hexane U**	66.8	3.0	29.37	0.12	3.15	0.03	364.1	40.5
**MC**	42.2	2.1	178.66	1.28	1.95	0.01	189.8	0.5
**EA**	15.8	1.5	8.80	0.09	5.03	0.06	969.2	5.0
**P Butanol**	17.8	0.9	12.96	0.32	4.37	0.02	792.1	4.7
**H Butanol**	15.1	1.2	32.53	0.41	2.83	0.04	427.1	0.8
**Water**	3.6	1.4	267.19	0.39	0.89	0.05	81.4	1.5

**Normality Test (Shapiro-Wilk):**	Passed (p = 0.228)	Passed (p = 0.228)	Passed (p = 0.813)
**Constant Variance Test:**	Passed (p = 0.537)	Passed (p = 0.537)	Passed (p = 0.102)
**Pearson Correlation:**	r = 0.794	r = -0.318	r = -0.431
	$p < 0.05$	p = 0.443	p = 0.286

CELL VIABILITY

%

Experiment	Control			LPS		
	RBE (μg/mL)					
	0	50	100	0	50	100
#1	118.6	104.9	96.5	124.1	119.9	106.0
#2	99.7	82.1	115.2	102.0	81.3	111.6
#3	81.6	84.7	90.2	105.7	95.4	94.4
#4	87.7	79.0	88.6	121.6	111.7	100.8
#5	108.4	107.6	111.8	108.1	125.6	91.1
#6	103.9	91.0	107.7	109.5	86.7	109.7
**mean**	**100.0**	**91.5**	**101.7**	**111.8**	**103.4**	**102.3**
**SD**	**13.6**	**12.1**	**11.4**	**9.0**	**18.3**	**8.3**
**SEM**	**5.5**	**4.9**	**4.7**	**3.7**	**7.5**	**3.4**
vs. control				0.112	0.111	0.934
vs. 0 ug/ml		0.441	0.818		0.443	0.482

Normality Test (Shapiro-Wilk): Passed (p = 0.395)

Equal Variance Test (Brown-Forsythe): Passed (p = 0.152)

Two-Way ANOVA

Source of Variation	DF	SS	MS	F	p
LPS	1	591.370	591.370	3.762	0.062
Dose	2	427.46	213.73	1.360	0.272
LPS x Dose	2	253.170	126.585	0.805	0.456
Residual	30	4715.7	157.19		
Total	35	5987.7	171.08		

CELL VIABILITY

%

Experiment	Control			LPS		
	Ethyl acetate (μg/mL)					
	0	50	100	0	50	100
#1	106.2	89.8	97.1	99.7	97.9	99.4
#2	96.4	102.1	108.0	91.0	102.2	106.2
#3	97.3	95.3	97.8	112.1	100.0	112.7
#4	107.2	116.2	105.8	106.8	103.1	95.8
#5	94.1	117.0	99.1	94.4	102.2	94.1
#6	98.7	104.6	95.1	97.0	95.4	106.5
**mean**	**100.0**	**104.2**	**100.5**	**100.2**	**100.1**	**102.5**
**SD**	**5.4**	**10.9**	**5.2**	**7.9**	**3.0**	**7.2**
**SEM**	**2.2**	**4.5**	**2.1**	**3.2**	**1.2**	**2.9**
vs. control				0.966	0.331	0.629
vs. 0 ug/ml		0.677	0.906		0.994	0.821

Normality Test (Shapiro-Wilk): Passed (p = 0.561)

Equal Variance Test (Brown-Forsythe): Passed (p = 0.171)

Two-Way ANOVA

Source of Variation	DF	SS	MS	F	p
LPS	1	3.464	3.464	0.070	0.794
Dose	2	26.817	13.408	0.269	0.766
LPS x Dose	2	57.291	28.646	0.574	0.569
Residual	30	######	49.876		
Total	35	1583.8	45.253		

CELL VIABILITY

%

Experiment	Control			LPS		
	n-Butanol (μg/mL)					
	0	50	100	0	50	100
#1	106.2	89.8	97.1	99.7	103.5	95.6
#2	96.4	102.1	108.0	91.0	100.4	102.0
#3	97.3	95.3	97.8	99.3	102.1	98.5
#4	107.2	116.2	105.8	102.4	94.5	95.0
#5	94.1	117.0	99.1	95.4	102.4	100.5
#6	98.7	104.6	95.1	95.2	97.3	96.1
**mean**	**100.0**	**104.2**	**100.5**	**97.2**	**100.1**	**98.0**
**SD**	**5.4**	**10.9**	**5.2**	**4.1**	**3.5**	**2.9**
**SEM**	**2.2**	**4.5**	**2.1**	**1.7**	**1.4**	**1.2**
vs. control				0.418	0.240	0.468
vs. 0 ug/ml		0.550	0.889		0.793	0.821

Normality Test (Shapiro-Wilk): Passed (p = 0.516)

Equal Variance Test (Brown-Forsythe): Passed (p = 0.065)

Two-Way ANOVA

Source of Variation	DF	SS	MS	F	p
LPS	1	89.675	89.675	2.529	0.122
Dose	2	85.028	42.514	1.199	0.316
LPS x Dose	2	4.288	2.144	0.061	0.941
Residual	30	######	35.455		
Total	35	######	35.504		

NO PRODUCTION

uM nitrite

Experiment	Control			LPS		
	RBE (µg/mL)					
	0	50	100	0	50	100
#1	0.2	0.6	2.1	60.2	42.9	27.9
#2	0.4	0.4	2.0	58.8	37.7	27.0
#3	0.3	0.5	1.1	59.3	41.8	22.2
#4	0.3	0.7	1.1	59.7	42.6	25.1
#5	0.3	0.5	1.1	60.2	43.9	27.1
#6	0.3	0.3	1.2	58.0	43.7	23.9
mean	0.3	0.5	1.4	59.4	42.1	25.5
SD	0.1	0.1	0.5	0.9	2.3	2.2
SEM	0.0	0.1	0.2	0.4	0.9	0.9

Logarithmic transformation

Experiment	Control			LPS		
	RBE (µg/mL)					
	0	50	100	0	50	100
#1	-0.74	-0.21	0.32	1.78	1.63	1.45
#2	-0.43	-0.37	0.29	1.77	1.58	1.43
#3	-0.59	-0.29	0.05	1.77	1.62	1.35
#4	-0.47	-0.16	0.05	1.78	1.63	1.40
#5	-0.50	-0.31	0.04	1.78	1.64	1.43
#6	-0.47	-0.52	0.06	1.76	1.64	1.38
**mean**	**-0.53**	**-0.31**	**0.14**	**1.77**	**1.62**	**1.41**
**SD**	**0.12**	**0.13**	**0.13**	**0.01**	**0.02**	**0.04**
**SEM**	**0.05**	**0.05**	**0.05**	**0.00**	**0.01**	**0.02**
vs. control				<0.001	<0.001	<0.001
vs. 0 µg/ml		<0.001	<0.001		0.008	<0.001
vs. 50 µg/ml			<0.001			<0.001

Normality Test (Shapiro-Wilk): Passed (p = 0.057)

Equal Variance Test (Brown-Forsythe): Passed (p = 0.162)

Two-Way ANOVA

Source of Variation	DF	SS	MS	*F*	*p*
LPS	1	30.359	30.359	######	<0.001
Dose	2	0.147	0.0737	8.809	<0.001
LPS x Dose	2	1.653	0.827	98.773	<0.001
Residual	30	0.251	0.0084		
Total	35	32.411	0.926		

NO PRODUCTION

uM nitrite

Experiment	Control			LPS		
	Ethyl acetate (µg/mL)					
	0	50	100	0	50	100
#1	0.2	0.4	1.7	52.3	45.2	44.5
#2	0.2	0.5	1.6	51.7	45.6	43.6
#3	0.3	0.9	2.5	54.6	52.0	45.8
#4	0.2	0.4	2.1	59.4	50.8	46.0
#5	0.2	0.8	2.7	59.7	50.7	48.0
mean	0.2	0.6	2.1	55.5	48.9	45.6
SD	0.0	0.2	0.5	3.8	3.2	1.7
SEM	0.0	0.1	0.2	1.6	1.3	0.7

Logarithmic transformation

Experiment	Control			LPS		
	Ethyl acetate (µg/mL)					
	0	50	100	0	50	100
#1	-0.66	-0.43	0.23	1.72	1.66	1.65
#2	-0.64	-0.28	0.20	1.71	1.66	1.64
#3	-0.56	-0.05	0.41	1.74	1.72	1.66
#4	-0.65	-0.36	0.32	1.77	1.71	1.66
#5	-0.82	-0.10	0.43	1.78	1.70	1.68
mean	-0.67	-0.24	0.32	1.74	1.69	1.66
SD	0.10	0.17	0.10	0.03	0.03	0.02
SEM	0.04	0.07	0.04	0.01	0.01	0.01
**median**	**-0.6**	**-0.3**	**0.3**	**1.7**	**1.7**	**1.7**
**Q1**	**-0.7**	**-0.4**	**0.2**	**1.7**	**1.7**	**1.6**
**Q3**	**-0.6**	**-0.1**	**0.4**	**1.8**	**1.7**	**1.7**
vs. control				<0.001	<0.001	<0.001
vs. 0 ug/ml		0.002	<0.001		0.040	0.004
vs. 50 µg/ml			<0.001			0.066

Normality Test (Shapiro-Wilk): Passed (p = 0.17[illegible]

Equal Variance Test (Brown-Forsythe): Failed (p < 0.050)

Two-factor linear model with heteroscedasticity-robust standard errors

Source of Variation	DF	*F*	*p*
LPS	1	2344.3	<0.001
Dose	2	98.60	<0.001
LPS x Dose	2	110.16	<0.001

NO PRODUCTION

uM nitrite

Experiment	Control			LPS		
	n-Butanol (µg/mL)					
	0	50	100	0	50	100
#1	0.2	0.5	2.6	52.3	44.0	41.7
#2	0.2	1.1	3.2	51.7	45.0	41.5
#3	0.3	0.6	2.3	54.6	51.8	47.4
#4	0.2	0.3	1.9	59.4	51.9	47.6
#5	0.2	0.8	2.4	59.7	52.0	44.5
mean	0.2	0.6	2.5	55.5	48.9	44.5
SD	0.0	0.3	0.5	3.8	4.1	3.0
SEM	0.0	0.1	0.2	1.6	1.7	1.2
**median**	**0.2**	**0.6**	**2.4**	**54.6**	**51.8**	**44.5**
**Q1**	**0.2**	**0.5**	**2.3**	**52.3**	**45.0**	**41.7**
**Q3**	**0.2**	**0.8**	**2.6**	**59.4**	**51.9**	**47.4**
vs. control				<0.001	<0.001	0.002
vs. 0 ug/ml		0.016	0.002		0.012	0.008
vs. 50 µg/ml			0.007			0.060

Normality Test (Shapiro-Wilk): Failed (p < 0.050)

Kruskal-Wallis Test:

H = 27.168 with 5 degrees of freedom (p =< 0.001)

Nos2 mRNA

fold vs. 0 ug/ml in control

Experiment	Control			LPS		
	RBE (µg/mL)					
	0	50	100	0	50	100
#1	1.1	1.2	1.3	105.9	75.3	24.4
#2	0.9	2.3	2.6	124.3	56.8	21.2
#3	0.9	1.8	2.1	75.5	65.4	15.2
#4	1.0	1.8	2.1	104.3	61.6	21.1
#5	1.1	2.0	1.9	102.9	60.2	20.8
#6	0.9	1.9	2.0	96.1	61.0	20.0
mean	1.0	1.8	2.0	101.5	63.4	20.4
SD	0.1	0.4	0.4	15.8	6.5	3.0
SEM	0.0	0.1	0.2	6.5	2.6	1.2
**median**	**1.0**	**1.9**	**2.0**	**103.6**	**61.3**	**21.0**
**Q1**	**0.9**	**1.8**	**1.9**	**97.8**	**60.4**	**20.2**
**Q3**	**1.1**	**2.0**	**2.1**	**105.5**	**64.4**	**21.1**
vs. control				<0.001	<0.001	<0.001
vs. 0 µg/ml		<0.001	0.003		0.004	<0.001
vs. 50 µg/ml			0.262			0.004

Normality Test (Shapiro-Wilk): Failed (p < 0.050)

Kruskal-Wallis Test:

H = 33.228 with 5 degrees of freedom (p =< 0.001)

Ptgs2 mRNA

fold vs. 0 ug/ml in control

Experiment	Control			LPS		
	RBE (µg/mL)					
	0	50	100	0	50	100
#1	0.9	0.8	0.9	19.1	9.4	3.9
#2	1.1	0.7	0.9	31.0	5.4	7.5
#3	1.0	0.8	0.9	23.0	7.1	5.4
#4	1.3	1.0	1.3	32.6	5.8	2.5
#5	0.8	0.9	0.8	20.3	2.7	1.1
#6	1.0	1.0	1.0	23.5	3.4	1.1
mean	1.0	0.9	1.0	24.9	5.6	3.6
SD	0.2	0.1	0.2	5.6	2.4	2.6
SEM	0.1	0.1	0.1	2.3	1.0	1.0

Logarithmic transformation

Experiment	Control			LPS		
	RBE (µg/mL)					
	0	50	100	0	50	100
#1	-0.03	-0.12	-0.03	1.28	0.97	0.59
#2	0.03	-0.17	-0.04	1.49	0.73	0.88
#3	0.00	-0.08	-0.03	1.36	0.85	0.73
#4	0.10	-0.02	0.11	1.51	0.76	0.39
#5	-0.12	-0.04	-0.12	1.31	0.43	0.06
#6	-0.01	0.01	-0.02	1.37	0.53	0.04
mean	0.00	-0.07	-0.02	1.39	0.71	0.45
SD	0.07	0.07	0.08	0.10	0.20	0.35
SEM	0.03	0.03	0.03	0.04	0.08	0.14
**median**	**-0.01**	**-0.06**	**-0.03**	**1.37**	**0.75**	**0.49**
**Q1**	**-0.03**	**-0.11**	**-0.04**	**1.32**	**0.58**	**0.14**
**Q3**	**0.02**	**-0.02**	**-0.02**	**1.46**	**0.83**	**0.70**
vs. control				<0.001	<0.001	0.041
vs. 0 ug/ml		0.274	0.702		<0.001	0.002
vs. 50 µg/ml			0.528			0.294

Normality Test (Shapiro-Wilk): Passed (p = 0.059)

Equal Variance Test (Brown-Forsythe): Failed (p < 0.050)

Two-factor linear model with heteroscedasticity-robust standard errors

Source of Variation	DF	F	p
LPS	1	688.90	<0.001
Dose	2	1.19	0.317
LPS x Dose	2	26.78	<0.001

Tnfa mRNA

fold vs. 0 ug/ml in control

Experiment	Control			LPS		
	RBE (µg/mL)					
	0	50	100	0	50	100
#1	0.96	1.19	1.10	6.72	5.02	1.81
#2	0.86	0.86	0.86	4.60	3.00	2.48
#3	1.17	1.07	0.98	4.74	3.05	2.48
#4	0.81	1.39	1.16	7.07	5.10	2.62
#5	1.10	1.22	1.06	5.07	3.45	2.23
#6	1.08	1.02	1.32	3.97	3.24	1.65
mean	1.00	1.12	1.08	5.36	3.81	2.21
SD	0.14	0.18	0.16	1.25	0.98	0.40
SEM	0.06	0.07	0.06	0.51	0.40	0.16

Logarithmic transformation

Experiment	Control			LPS		
	RBE (µg/mL)					
	0	50	100	0	50	100
#1	-0.02	0.07	0.04	0.83	0.70	0.26
#2	-0.06	-0.07	-0.06	0.66	0.48	0.39
#3	0.07	0.03	-0.01	0.68	0.48	0.40
#4	-0.09	0.14	0.07	0.85	0.71	0.42
#5	0.04	0.09	0.03	0.71	0.54	0.35
#6	0.03	0.01	0.12	0.60	0.51	0.22
**mean**	**0.00**	**0.05**	**0.03**	**0.72**	**0.57**	**0.34**
**SD**	**0.06**	**0.07**	**0.06**	**0.10**	**0.11**	**0.08**
**SEM**	**0.03**	**0.03**	**0.03**	**0.04**	**0.04**	**0.03**
vs. control				<0.001	<0.001	<0.001
vs. 0 µg/ml		0.661	0.695		0.003	<0.001
vs. 50 µg/ml			0.782			<0.001

Normality Test (Shapiro-Wilk): Passed (p = 0.250)

Equal Variance Test (Brown-Forsythe): Passed (p = 0.845)

Two-Way ANOVA

Source of Variation	DF	SS	MS	F	p
LPS	1	2.434	2.434	357.066	<0.001
Dose	2	0.189	0.0943	13.834	<0.001
LPS x Dose	2	0.263	0.131	19.266	<0.001
Residual	30	0.204	0.0068		
Total	35	3.089	0.0883		

Il1b mRNA

fold vs. 0 ug/ml in control

Experiment	Control			LPS		
	RBE (µg/mL)					
	0	50	100	0	50	100
#1	1.1	1.0	1.1	83.9	8.2	0.9
#2	1.0	1.0	0.9	50.6	5.5	2.1
#3	0.9	0.6	0.7	65.2	6.7	1.4
#4	1.3	1.3	1.1	85.9	6.7	1.6
#5	0.7	0.6	0.9	65.5	6.0	0.8
#6	1.0	1.0	0.6	49.9	6.6	1.2
mean	1.0	0.9	0.9	66.8	6.6	1.3
SD	0.2	0.2	0.2	15.5	0.9	0.5
SEM	0.1	0.1	0.1	6.3	0.4	0.2

Logarithmic transformation

Experiment	Control			LPS		
	RBE (µg/mL)					
	0	50	100	0	50	100
#1	0.05	-0.01	0.04	1.92	0.92	-0.03
#2	0.01	-0.02	-0.03	1.70	0.74	0.32
#3	-0.07	-0.23	-0.14	1.81	0.83	0.14
#4	0.11	0.10	0.04	1.93	0.83	0.22
#5	-0.17	-0.19	-0.06	1.82	0.78	-0.11
#6	0.01	0.00	-0.24	1.70	0.82	0.07
**mean**	**-0.01**	**-0.06**	**-0.07**	**1.82**	**0.82**	**0.10**
**SD**	**0.10**	**0.13**	**0.11**	**0.10**	**0.06**	**0.16**
**SEM**	**0.04**	**0.05**	**0.04**	**0.04**	**0.02**	**0.06**
vs. control				<0.001	<0.001	0.014
vs. 0 µg/ml		0.702	0.757		<0.001	<0.001
vs. 50 µg/ml			NS			<0.001

Normality Test (Shapiro-Wilk): Passed (p = 0.470)

Equal Variance Test (Brown-Forsythe): Passed (p = 0.463)

Two-Way ANOVA

Source of Variation	DF	SS	MS	F	p
LPS	1	8.225	8.225	657.452	<0.001
Dose	2	4.759	2.379	190.192	<0.001
LPS x Dose	2	4.139	2.070	165.44	<0.001
Residual	30	0.375	0.0125		
Total	35	17.498	0.500		

Il6 mRNA

fold vs. 0 ug/ml in control

Experiment	Control			LPS		
	RBE (µg/mL)					
	0	50	100	0	50	100
#1	0.8	0.8	0.8	458.1	152.6	80.6
#2	1.1	0.8	1.1	333.5	155.3	71.9
#3	1.0	1.0	1.1	294.5	188.2	41.5
#4	0.8	0.9	1.0	362.7	165.7	80.8
#5	1.0	0.8	1.0	414.4	177.6	77.2
#6	1.1	1.3	0.9	318.0	116.1	75.3
mean	1.0	0.9	1.0	363.5	159.2	71.2
SD	0.1	0.2	0.1	62.1	25.1	14.9
SEM	0.1	0.1	0.0	25.3	10.2	6.1

Logarithmic transformation

Experiment	Control			LPS		
	RBE (µg/mL)					
	0	50	100	0	50	100
#1	-0.08	-0.08	-0.11	2.66	2.18	1.91
#2	0.06	-0.10	0.02	2.52	2.19	1.86
#3	0.01	0.00	0.04	2.47	2.27	1.62
#4	-0.07	-0.05	0.01	2.56	2.22	1.91
#5	0.02	-0.07	0.02	2.62	2.25	1.89
#6	0.05	0.11	-0.03	2.50	2.06	1.88
**mean**	**0.00**	**-0.03**	**-0.01**	**2.56**	**2.20**	**1.84**
**SD**	**0.06**	**0.08**	**0.06**	**0.07**	**0.07**	**0.11**
**SEM**	**0.02**	**0.03**	**0.02**	**0.03**	**0.03**	**0.05**
vs. control				<0.001	<0.001	<0.001
vs. 0 µg/ml		0.895	0.884		<0.001	<0.001
vs. 50 µg/ml			NS			<0.001

Normality Test (Shapiro-Wilk): Passed (p = 0.140)

Equal Variance Test (Brown-Forsythe): Passed (p = 0.990)

Two-Way ANOVA

Source of Variation	DF	SS	MS	F	p
LPS	1	44.094	44.094	######	<0.001
Dose	2	0.779	0.389	64.654	<0.001
LPS x Dose	2	0.750	0.375	62.278	<0.001
Residual	30	0.181	0.006		
Total	35	45.803	1.309		

CELL VIABILITY

%

Experiment	Control				LPS			
	Ellagic acid (μM)							
	0	10	30	100	0	10	30	100
#1	102.6	94.6	102.7	95.0	105.4	117.2	93.8	99.5
#2	82.0	112.0	84.7	86.3	97.4	107.5	110.4	87.7
#3	115.4	90.9	87.0	93.6	106.6	105.1	116.4	95.5
#4	101.1	101.1	104.9	100.5	99.3	84.5	96.3	88.0
#5	94.4	94.4	108.0	84.6	90.6	94.2	107.3	109.6
#6	104.5	104.5	110.4	80.0	83.5	85.8	101.0	97.0
**mean**	**100.0**	**99.6**	**99.6**	**90.0**	**97.1**	**99.1**	**104.2**	**96.2**
**SD**	**11.1**	**7.8**	**11.0**	**7.6**	**8.9**	**13.0**	**8.7**	**8.1**
**SEM**	**4.5**	**3.2**	**4.5**	**3.1**	**3.6**	**5.3**	**3.5**	**3.3**
vs. control					0.612	0.927	0.417	0.275
vs. 0 uM		1.000	0.997	0.401		0.928	0.701	0.868

Normality Test (Shapiro-Wilk): Passed (p = 0.523)

Equal Variance Test (Brown-Forsythe): Passed (p = 0.823)

Two-Way ANOVA

Source of Variation	DF	SS	MS	*F*	*p*
LPS	1	41.285	41.285	0.438	0.512
Does	3	494.75	164.92	1.748	0.173
LPS x Does	3	163.15	54.385	0.577	0.634
Residual	40	3773.3	94.333		
Total	47	4472.5	95.159		

CELL VIABILITY

%

Experiment	Control				LPS			
	Ethyl gallate (μM)							
	0	10	30	100	0	10	30	100
#1	116.4	92.2	104.2	103.5	98.5	97.8	90.6	96.0
#2	97.5	94.7	103.4	128.0	88.8	97.9	89.8	91.1
#3	86.1	85.0	99.5	98.2	98.0	104.5	90.3	92.2
#4	101.1	101.1	110.3	109.6	97.7	93.0	105.9	96.8
#5	94.4	94.4	108.0	84.6	99.7	103.6	104.3	120.5
#6	104.5	104.5	110.4	80.0	91.8	94.4	111.1	106.7
**mean**	**100.0**	**95.3**	**106.0**	**100.7**	**95.7**	**98.5**	**98.7**	**100.5**
**SD**	**10.2**	**6.9**	**4.3**	**17.5**	**4.4**	**4.7**	**9.5**	**11.2**
**SEM**	**4.2**	**2.8**	**1.8**	**7.1**	**1.8**	**1.9**	**3.9**	**4.6**
vs. control					0.445	0.562	0.194	0.982
vs. 0 uM		0.642	0.814	0.905		0.978	0.989	0.949

Normality Test (Shapiro-Wilk): Passed (p = 0.298)

Equal Variance Test (Brown-Forsythe): Passed (p = 0.074)

Two-Way ANOVA

Source of Variation	DF	SS	MS	*F*	*p*
LPS	1	53.740	53.740	0.585	0.449
Does	3	222.08	74.026	0.806	0.498
LPS x Does	3	192.72	64.241	0.700	0.558
Residual	40	3673.4	91.834		
Total	47	4141.9	88.126		

CELL VIABILITY

%

Experiment	Control				LPS			
	Epicatechin (μM)							
	0	10	30	100	0	10	30	100
#1	111.2	108.1	115.0	109.7	106.1	103.8	107.2	103.3
#2	93.4	114.7	104.7	102.0	96.0	105.9	99.5	102.5
#3	95.4	102.5	106.0	98.3	93.9	105.8	96.0	102.3
#4	105.1	98.4	91.4	92.1	93.5	101.9	115.5	104.8
#5	90.8	103.5	87.1	88.5	90.8	98.8	95.1	100.6
#6	104.0	99.8	87.8	92.3	105.3	91.8	86.1	98.3
**mean**	**100.0**	**104.5**	**98.6**	**97.2**	**97.6**	**101.3**	**99.9**	**102.0**
**SD**	**8.0**	**6.0**	**11.5**	**7.8**	**6.5**	**5.4**	**10.3**	**2.3**
**SEM**	**3.3**	**2.5**	**4.7**	**3.2**	**2.6**	**2.2**	**4.2**	**0.9**
vs. control					0.593	0.479	0.780	0.287
vs. 0 uM		0.781	0.762	0.894		0.926	0.977	0.912

Normality Test (Shapiro-Wilk): Passed (p = 0.710)

Equal Variance Test (Brown-Forsythe): Passed (p = 0.054)

Two-Way ANOVA

Source of Variation	DF	SS	MS	*F*	*p*
LPS	1	0.175	0.175	0.00295	0.957
Does	3	128	42.666	0.719	0.546
LPS x Does	3	121.23	40.411	0.681	0.569
Residual	40	2373.3	59.332		
Total	47	2622.7	55.802		

NO PRODUCTION

uM nitrite

Experiment	Control				LPS			
	Ellagic acid (μM)							
	0	10	30	100	0	10	30	100
#1	0.2	0.4	0.1	1.0	50.7	48.6	38.8	22.9
#2	0.4	0.1	0.4	0.9	52.9	50.0	41.1	25.0
#3	0.3	0.3	0.3	1.0	54.2	51.6	38.1	18.8
#4	0.6	0.5	0.5	1.2	58.5	48.9	28.2	21.9
#5	0.3	0.2	0.2	0.8	55.9	50.5	25.8	21.4
#6	0.3	0.2	0.1	1.0	54.2	46.2	33.3	15.5
mean	0.3	0.3	0.3	1.0	54.4	49.3	34.2	20.9
SD	0.1	0.1	0.2	0.1	2.7	1.9	6.2	3.3
SEM	0.1	0.1	0.1	0.0	1.1	0.8	2.5	1.4
**median**	**0.3**	**0.2**	**0.2**	**1.0**	**54.2**	**49.5**	**35.7**	**21.7**
**Q1**	**0.3**	**0.2**	**0.1**	**0.9**	**53.2**	**48.7**	**29.4**	**19.4**
**Q3**	**0.4**	**0.4**	**0.4**	**1.0**	**55.5**	**50.4**	**38.6**	**22.7**
vs. control					<0.001	<0.001	<0.001	0.004
vs. 0 uM		0.560	0.139	<0.001		0.006	<0.001	<0.001
vs. 10 uM			0.968	0.007			0.003	<0.001
vs. 30 uM				<0.001				0.004

Normality Test (Shapiro-Wilk): Failed (p < 0.050)

Kruskal-Wallis Test: H = 44.224 with 7 degrees of freedom (p =< 0.001)

NO PRODUCTION

uM nitrite

Experiment	Control				LPS			
	Ethyl gallate (μM)							
	0	10	30	100	0	10	30	100
#1	0.2	0.2	0.4	0.5	50.0	44.8	29.8	9.9
#2	0.2	0.2	0.1	0.5	52.6	47.4	34.4	12.8
#3	0.3	0.2	0.3	0.7	53.5	46.6	37.5	11.6
#4	0.2	0.1	0.2	0.9	62.2	47.3	30.2	9.0
#5	0.2	0.2	0.1	0.5	65.2	49.3	28.3	16.8
#6	0.3	0.2	0.1	0.5	55.9	45.1	25.2	13.0
mean	0.2	0.2	0.2	0.6	56.5	46.8	30.9	12.2
SD	0.0	0.1	0.1	0.2	5.9	1.7	4.4	2.8
SEM	0.0	0.0	0.1	0.1	2.4	0.7	1.8	1.1
**median**	**0.2**	**0.2**	**0.2**	**0.5**	**54.7**	**46.9**	**30.0**	**12.2**
**Q1**	**0.2**	**0.2**	**0.1**	**0.5**	**52.8**	**45.4**	**28.7**	**10.4**
**Q3**	**0.3**	**0.2**	**0.3**	**0.6**	**60.6**	**47.4**	**33.4**	**12.9**
vs. control					<0.001	<0.001	<0.001	0.004
vs. 0 uM		0.427	0.161	<0.001		0.004	<0.001	<0.001
vs. 10 uM			0.660	0.005			0.004	<0.001
vs. 30 uM				<0.001				0.004

Normality Test (Shapiro-Wilk): Failed (p < 0.050)

Kruskal-Wallis Test: H = 44.334 with 7 degrees of freedom (p =< 0.001)

NO PRODUCTION

uM nitrite

Experiment	Control				LPS			
	Epicatechin (μM)							
	0	10	30	100	0	10	30	100
#1	0.2	0.1	0.3	1.1	61.5	58.5	56.1	31.3
#2	0.4	0.4	0.3	1.5	66.0	62.8	58.2	31.4
#3	0.3	0.4	0.3	1.2	65.0	61.9	59.5	29.9
#4	0.3	0.3	0.4	0.7	49.3	36.5	29.1	16.3
#5	0.5	0.2	0.2	0.5	45.7	39.2	26.0	10.2
#6	0.3	0.1	0.2	0.5	32.1	25.4	19.2	9.5
mean	0.3	0.3	0.3	0.9	53.3	47.4	41.3	21.4
SD	0.1	0.2	0.1	0.4	13.3	15.7	18.5	10.6
SEM	0.0	0.1	0.0	0.2	5.4	6.4	7.5	4.3

Logarithmic transformation

Experiment	Control				LPS			
	Epicatechin (μM)							
	0	10	30	100	0	10	30	100
#1	-0.63	-1.22	-0.47	0.06	1.79	1.77	1.75	1.50
#2	-0.41	-0.41	-0.60	0.17	1.82	1.80	1.76	1.50
#3	-0.51	-0.37	-0.59	0.07	1.81	1.79	1.77	1.48
#4	-0.47	-0.50	-0.39	-0.15	1.69	1.56	1.46	1.21
#5	-0.33	-0.70	-0.63	-0.31	1.66	1.59	1.41	1.01
#6	-0.50	-0.97	-0.68	-0.28	1.51	1.40	1.28	0.98
mean	-0.47	-0.69	-0.56	-0.07	1.71	1.65	1.57	1.28
SD	0.10	0.34	0.11	0.20	0.12	0.16	0.21	0.25
SEM	0.04	0.14	0.04	0.08	0.05	0.07	0.09	0.10
**median**	**-0.48**	**-0.60**	**-0.59**	**-0.05**	**1.74**	**1.68**	**1.61**	**1.34**
**Q1**	**-0.51**	**-0.90**	**-0.62**	**-0.25**	**1.67**	**1.57**	**1.43**	**1.06**
**Q3**	**-0.42**	**-0.43**	**-0.50**	**0.07**	**1.81**	**1.79**	**1.76**	**1.49**
vs. control					<0.001	<0.001	<0.001	<0.001
vs. 0 uM		0.360	0.382	0.009		0.942	0.588	0.017
vs. 10 uM			0.778	0.014			0.968	0.041
vs. 30 uM				0.003				0.108

Normality Test (Shapiro-Wilk): Passed (p = 0.153)

Equal Variance Test (Brown-Forsythe): Failed (p < 0.050)

Two-factor linear model with heteroscedasticity-robust standard errors

Source of Variation	DF	F	p
LPS	1	977.44	<0.001
Dose	3	8.37	<0.001
LPS x Dose	3	10.40	<0.001

Nos2 mRNA

fold vs. 0 ug/ml in control

Experiment	Control				LPS			
	Ellagic acid (μM)							
	0	10	30	100	0	10	30	100
#1	1.0	0.9	1.2	1.8	98.2	66.2	87.0	27.1
#2	0.7	0.9	0.5	1.1	119.3	87.1	60.4	26.5
#3	1.2	1.4	1.1	1.7	120.5	99.1	74.4	57.1
#4	1.1	0.7	0.9	1.8	122.3	67.5	84.5	30.6
#5	1.1	0.9	0.8	1.4	114.9	80.5	70.1	45.4
#6	0.8	1.5	1.0	1.2	86.7	105.1	78.0	30.0
#7	1.5	1.0	1.2	1.5	109.1	67.9	77.8	37.2
#8	0.8	0.9	0.5	1.8	119.9	83.8	82.9	29.4
#9	0.7	1.3	0.9	1.2	90.8	97.2	54.5	48.2
mean	1.0	1.1	0.9	1.5	109.1	83.8	74.4	36.8
SD	0.3	0.3	0.3	0.3	13.8	14.6	11.0	10.9
SEM	0.1	0.1	0.1	0.1	4.6	4.9	3.7	3.6

Logarithmic translation

Experiment	Control				LPS			
	Ellagic acid (μM)							
	0	10	30	100	0	10	30	100
#1	0.02	-0.03	0.09	0.26	1.99	1.82	1.94	1.43
#2	-0.15	-0.05	-0.30	0.06	2.08	1.94	1.78	1.42
#3	0.10	0.15	0.05	0.24	2.08	2.00	1.87	1.76
#4	0.02	-0.13	-0.02	0.26	2.09	1.83	1.93	1.49
#5	0.04	-0.07	-0.07	0.15	2.06	1.91	1.85	1.66
#6	-0.07	0.18	-0.01	0.07	1.94	2.02	1.89	1.48
#7	0.19	0.00	0.09	0.17	2.04	1.83	1.89	1.57
#8	-0.10	-0.03	-0.28	0.25	2.08	1.92	1.92	1.47
#9	-0.17	0.11	-0.04	0.08	1.96	1.99	1.74	1.68
**mean**	**-0.01**	**0.02**	**-0.06**	**0.17**	**2.03**	**1.92**	**1.87**	**1.55**
**SD**	**0.12**	**0.11**	**0.14**	**0.09**	**0.06**	**0.08**	**0.07**	**0.12**
**SEM**	**0.04**	**0.04**	**0.05**	**0.03**	**0.02**	**0.03**	**0.02**	**0.04**
vs. control					<0.001	<0.001	<0.001	<0.001
vs. 0 μM		0.518	0.631	0.001		0.033	0.002	<0.001
vs. 10 μM			0.355	0.008			0.295	<0.001
vs. 30 μM				<0.001				<0.001

Normality Test (Shapiro-Wilk): Passed (p = 0.668)
Equal Variance Test (Brown-Forsythe): Passed (p = 0.417)
Two-Way ANOVA

Source of Variation	DF	SS	MS	*F*	*p*
LPS	1	59.158	59.158	5791.780	<0.001
Dose	3	0.235	0.0783	7.664	<0.001
LPS x Dose	3	1.182	0.394	38.754	<0.001
Residual	64	0.654	0.0102		
Total	71	61.229	0.862		

Nos2 mRNA

fold vs. 0 ug/ml in control

Experiment	Control				LPS			
	Ethyl gallate (μM)							
	0	10	30	100	0	10	30	100
#1	0.9	0.6	0.6	1.5	94.7	77.6	29.2	21.9
#2	0.8	1.0	0.8	1.7	112.7	64.6	53.1	25.3
#3	1.3	0.9	1.0	2.1	105.3	89.3	67.9	22.5
#4	0.6	1.4	0.9	1.9	94.0	91.9	39.9	20.7
#5	1.1	0.4	0.7	1.7	132.5	86.3	59.5	29.2
#6	1.3	0.9	0.9	2.0	88.1	66.2	48.7	19.2
#7	1.0	1.1	0.9	1.5	96.6	84.3	52.0	26.0
#8	1.0	0.8	0.8	2.0	95.4	63.1	45.3	19.2
#9	1.1	0.9	0.8	2.2	111.4	87.2	54.8	25.9
mean	1.0	0.9	0.8	1.8	103.4	79.0	50.0	23.3
SD	0.2	0.3	0.1	0.3	13.7	11.4	11.2	3.5
SEM	0.1	0.1	0.0	0.1	4.6	3.8	3.7	1.2
**median**	**1.0**	**0.9**	**0.8**	**1.9**	**96.0**	**85.3**	**50.4**	**23.3**
**Q1**	**1.0**	**0.8**	**0.8**	**1.7**	**94.4**	**70.7**	**46.1**	**19.6**
**Q3**	**1.1**	**1.0**	**0.9**	**2.0**	**107.7**	**87.0**	**54.1**	**26.0**
vs. control					<0.001	<0.001	<0.001	<0.001
vs. 0 μM		0.112	0.082	<0.001		<0.001	<0.001	<0.001
vs. 10 μM			0.112	<0.001			<0.001	<0.001
vs. 30 μM				<0.001				<0.001

Normality Test (Shapiro-Wilk): Failed (p < 0.050)
Kruskal-Wallis Test: H = 67.031 with 7 degrees of freedom (p =< 0.001)

Nos2 mRNA

fold vs. 0 ug/ml in control

Experiment	Control				LPS			
	Epicatechin (μM)							
	0	10	30	100	0	10	30	100
#1	0.7	0.6	1.4	1.3	76.4	87.8	114.7	89.1
#2	0.9	1.2	1.0	1.6	114.6	101.9	92.3	37.6
#3	1.4	1.2	1.2	2.0	113.2	115.0	107.9	61.0
#4	1.3	0.9	1.5	1.6	110.1	108.2	117.3	80.9
#5	0.9	1.4	1.0	1.5	81.3	111.9	92.7	51.6
#6	0.8	1.0	1.2	1.8	114.9	74.7	71.1	58.3
#7	0.9	1.0	0.8	1.7	116.1	99.7	89.5	58.5
#8	0.9	0.8	1.7	1.5	89.3	116.2	107.2	76.2
#9	1.1	1.2	1.2	1.6	96.2	115.2	105.9	55.6
mean	1.0	1.0	1.2	1.6	101.3	103.4	99.8	63.2
SD	0.3	0.2	0.3	0.2	15.8	14.2	14.7	16.0
SEM	0.1	0.1	0.1	0.1	5.3	4.7	4.9	5.3

Logarithmic translation

Experiment	Control				LPS			
	Epicatechin (μM)							
	0	10	30	100	0	10	30	100
#1	-0.16	-0.19	0.16	0.13	1.88	1.94	2.06	1.95
#2	-0.06	0.09	-0.01	0.19	2.06	2.01	1.97	1.58
#3	0.16	0.08	0.08	0.29	2.05	2.06	2.03	1.79
#4	0.13	-0.05	0.19	0.20	2.04	2.03	2.07	1.91
#5	-0.06	0.14	0.00	0.18	1.91	2.05	1.97	1.71
#6	-0.10	0.00	0.08	0.25	2.06	1.87	1.85	1.77
#7	-0.04	0.00	-0.11	0.24	2.06	2.00	1.95	1.77
#8	-0.02	-0.07	0.22	0.17	1.95	2.07	2.03	1.88
#9	0.06	0.08	0.10	0.21	1.98	2.06	2.02	1.75
mean	-0.01	0.01	0.08	0.20	2.00	2.01	1.99	1.79
SD	0.11	0.10	0.11	0.05	0.07	0.06	0.07	0.11
SEM	0.04	0.03	0.04	0.02	0.02	0.02	0.02	0.04
**median**	**-0.03**	**0.00**	**0.09**	**0.20**	**2.01**	**2.04**	**2.00**	**1.77**
**Q1**	**-0.05**	**-0.04**	**0.02**	**0.18**	**1.96**	**2.01**	**1.96**	**1.75**
**Q3**	**0.04**	**0.06**	**0.17**	**0.23**	**2.06**	**2.06**	**2.03**	**1.85**
vs. control					<0.001	<0.001	<0.001	<0.001
vs. 0 μM		0.639	0.102	<0.001		0.967	0.885	<0.001
vs. 10 μM			0.186	<0.001			0.975	<0.001
vs. 30 μM				0.013				<0.001

Normality Test (Shapiro-Wilk): Passed (p = 0.485)
Equal Variance Test (Brown-Forsythe): Passed (p = 0.450)
Two-Way ANOVA

Source of Variation	DF	SS	MS	*F*	*p*
LPS	1	63.540	63.540	8103.393	<0.001
Dose	3	0.0201	0.00670	0.855	0.469
LPS x Dose	3	0.550	0.183	23.395	<0.001
Residual	64	0.502	0.0078		
Total	71	64.612	0.910		

Ptgs2 mRNA

fold vs. 0 ug/ml in control

Experiment	Control				LPS			
	Ellagic acid (μM)							
	0	10	30	100	0	10	30	100
#1	1.2	1.2	3.2	3.6	24.6	18.4	21.8	6.8
#2	0.7	1.3	2.1	1.8	27.4	24.2	15.1	6.6
#3	1.1	1.9	3.5	4.4	32.7	27.6	18.6	14.3
#4	1.1	1.2	2.5	4.5	32.8	18.6	21.0	7.6
#5	0.8	1.4	1.4	3.3	26.1	22.2	17.4	11.3
#6	1.1	1.1	3.0	3.7	21.5	29.0	19.4	7.4
#7	1.5	1.1	2.2	3.4	27.7	19.2	19.8	9.5
#8	1.1	0.9	1.6	3.0	30.5	23.7	21.1	7.5
#9	0.4	1.0	1.6	2.6	23.1	27.5	13.8	12.3
mean	1.0	1.2	2.3	3.4	27.4	23.4	18.7	9.2
SD	0.3	0.3	0.8	0.8	4.0	4.1	2.7	2.8
SEM	0.1	0.1	0.3	0.3	1.3	1.4	0.9	0.9

Logarithmic translation

Experiment	Control				LPS			
	Ellagic acid (μM)							
	0	10	30	100	0	10	30	100
#1	0.09	0.07	0.51	0.56	1.39	1.26	1.34	0.83
#2	-0.15	0.11	0.31	0.26	1.44	1.38	1.18	0.82
#3	0.02	0.27	0.54	0.64	1.51	1.44	1.27	1.16
#4	0.04	0.08	0.40	0.65	1.52	1.27	1.32	0.88
#5	-0.11	0.16	0.13	0.52	1.42	1.35	1.24	1.05
#6	0.05	0.05	0.47	0.56	1.33	1.46	1.29	0.87
#7	0.18	0.03	0.34	0.53	1.44	1.28	1.30	0.98
#8	0.03	-0.03	0.19	0.47	1.48	1.37	1.32	0.87
#9	-0.37	0.01	0.21	0.41	1.36	1.44	1.14	1.09
**mean**	**-0.02**	**0.08**	**0.35**	**0.51**	**1.43**	**1.36**	**1.27**	**0.95**
**SD**	**0.16**	**0.09**	**0.15**	**0.12**	**0.06**	**0.08**	**0.07**	**0.12**
**SEM**	**0.05**	**0.03**	**0.05**	**0.04**	**0.02**	**0.03**	**0.02**	**0.04**
vs. control					<0.001	<0.001	<0.001	<0.001
vs. 0 μM		0.048	<0.001	<0.001		0.187	0.007	<0.001
vs. 10 μM			<0.001	<0.001			0.141	<0.001
vs. 30 μM				0.004				<0.001

Normality Test (Shapiro-Wilk): Passed (p = 0.268)

Equal Variance Test (Brown-Forsythe): Passed (p = 0.292)

Two-Way ANOVA

Source of Variation	DF	SS	MS	*F*	*p*
LPS	1	18.872	18.872	1507.599	<0.001
Dose	3	0.107	0.0357	2.852	0.044
LPS x Dose	3	2.735	0.912	72.829	<0.001
Residual	64	0.801	0.0125		
Total	71	22.515	0.317		

Ptgs2 mRNA

fold vs. 0 ug/ml in control

Experiment	Control				LPS			
	Ethyl gallate (μM)							
	0	10	30	100	0	10	30	100
#1	0.4	1.6	2.0	1.6	16.6	16.0	8.8	4.9
#2	0.8	1.8	2.3	2.0	24.5	18.9	9.5	7.0
#3	1.8	2.2	2.6	2.4	29.1	20.0	14.4	6.8
#4	1.0	1.6	3.0	3.0	20.4	15.4	11.1	6.0
#5	0.8	2.9	4.8	2.6	20.9	10.8	12.7	5.0
#6	1.1	1.5	2.6	3.8	26.0	13.4	12.6	7.0
#7	1.1	2.7	4.6	4.6	21.9	13.1	9.3	3.4
#8	0.7	2.1	3.5	4.1	23.3	10.9	8.5	3.0
#9	1.1	2.6	4.0	4.8	24.9	12.2	9.2	3.2
mean	1.0	2.1	3.3	3.2	23.1	14.5	10.7	5.1
SD	0.4	0.5	1.0	1.1	3.6	3.3	2.1	1.7
SEM	0.1	0.2	0.3	0.4	1.2	1.1	0.7	0.6

Logarithmic translation

Experiment	Control				LPS			
	Ethyl gallate (μM)							
	0	10	30	100	0	10	30	100
#1	-0.41	0.20	0.30	0.21	1.22	1.21	0.95	0.69
#2	-0.07	0.24	0.37	0.31	1.39	1.28	0.98	0.85
#3	0.25	0.34	0.42	0.38	1.46	1.30	1.16	0.83
#4	0.01	0.20	0.48	0.48	1.31	1.19	1.05	0.78
#5	-0.08	0.46	0.68	0.42	1.32	1.04	1.10	0.70
#6	0.05	0.18	0.42	0.58	1.42	1.13	1.10	0.84
#7	0.06	0.43	0.66	0.66	1.34	1.12	0.97	0.54
#8	-0.13	0.33	0.55	0.61	1.37	1.04	0.93	0.48
#9	0.04	0.42	0.60	0.68	1.40	1.09	0.96	0.50
**mean**	**-0.03**	**0.31**	**0.50**	**0.48**	**1.36**	**1.15**	**1.02**	**0.69**
**SD**	**0.18**	**0.11**	**0.13**	**0.16**	**0.07**	**0.10**	**0.08**	**0.15**
**SEM**	**0.06**	**0.04**	**0.04**	**0.05**	**0.02**	**0.03**	**0.03**	**0.05**
vs. control					<0.001	<0.001	<0.001	0.001
vs. 0 μM		<0.001	<0.001	<0.001		0.002	<0.001	<0.001
vs. 10 μM			0.009	0.013			0.033	<0.001
vs. 30 μM				0.778				<0.001

Normality Test (Shapiro-Wilk): Passed (p = 0.542)

Equal Variance Test (Brown-Forsythe): Passed (p = 0.115)

Two-Way ANOVA

Source of Variation	DF	SS	MS	*F*	*p*
LPS	1	9.855	9.855	596.071	<0.001
Dose	3	0.327	0.109	6.590	<0.001
LPS x Dose	3	3.423	1.141	69.017	<0.001
Residual	64	1.058	0.0165		
Total	71	14.664	0.207		

Ptgs2 mRNA

fold vs. 0 ug/ml in control

Experiment	Control				LPS			
	Epicatechin (μM)							
	0	10	30	100	0	10	30	100
#1	0.8	1.1	2.3	1.4	24.3	21.4	16.4	9.5
#2	0.7	2.0	1.3	1.1	25.1	15.4	25.0	14.4
#3	1.5	1.7	1.3	2.7	27.7	29.2	19.3	20.4
#4	0.8	2.7	2.1	2.6	25.8	26.9	13.3	12.2
#5	0.9	1.8	3.5	1.1	23.3	24.7	13.6	9.4
#6	1.2	2.5	2.4	1.6	26.7	16.5	13.0	6.9
#7	1.2	1.2	1.6	1.8	29.1	21.3	18.5	14.9
#8	1.0	1.2	1.7	2.4	31.7	14.8	16.1	10.8
#9	0.8	2.0	1.3	1.8	24.7	22.8	13.3	10.6
mean	1.0	1.8	1.9	1.8	26.5	21.4	16.5	12.1
SD	0.3	0.5	0.7	0.6	2.7	5.1	4.0	4.0
SEM	0.1	0.2	0.2	0.2	0.9	1.7	1.3	1.3

Logarithmic translation

Experiment	Control				LPS			
	Epicatechin (μM)							
	0	10	30	100	0	10	30	100
#1	-0.12	0.06	0.36	0.15	1.39	1.33	1.21	0.98
#2	-0.15	0.31	0.12	0.03	1.40	1.19	1.40	1.16
#3	0.19	0.24	0.12	0.43	1.44	1.47	1.29	1.31
#4	-0.09	0.43	0.32	0.42	1.41	1.43	1.12	1.08
#5	-0.02	0.26	0.54	0.05	1.37	1.39	1.13	0.97
#6	0.10	0.39	0.38	0.19	1.43	1.22	1.11	0.84
#7	0.06	0.09	0.22	0.24	1.46	1.33	1.27	1.17
#8	0.02	0.08	0.23	0.39	1.50	1.17	1.21	1.04
#9	-0.09	0.31	0.10	0.26	1.39	1.36	1.12	1.03
**mean**	**-0.01**	**0.24**	**0.26**	**0.24**	**1.42**	**1.32**	**1.21**	**1.06**
**SD**	**0.11**	**0.14**	**0.15**	**0.15**	**0.04**	**0.11**	**0.10**	**0.14**
**SEM**	**0.04**	**0.05**	**0.05**	**0.05**	**0.01**	**0.04**	**0.03**	**0.05**
vs. control					<0.001	<0.001	<0.001	<0.001
vs. 0 μM		<0.001	<0.001	<0.001		0.080	0.001	<0.001
vs. 10 μM			0.964	0.988			0.102	<0.001
vs. 30 μM				0.898				0.043

Normality Test (Shapiro-Wilk): Passed (p = 0.686)

Equal Variance Test (Brown-Forsythe): Passed (p = 0.088)

Two-Way ANOVA

Source of Variation	DF	SS	MS	*F*	*p*
LPS	1	20.619	20.619	1413.571	<0.001
Dose	3	0.155	0.0517	3.546	0.019
LPS x Dose	3	0.944	0.315	21.577	<0.001
Residual	64	0.934	0.0146		
Total	71	22.652	0.319		

Tnfa mRNA

fold vs. 0 ug/ml in control

Experiment	Control				LPS			
	Ellagic acid (µM)							
	0	10	30	100	0	10	30	100
#1	1.11	1.05	1.56	1.31	4.36	2.31	2.62	1.84
#2	0.80	0.84	0.90	1.37	4.96	2.20	2.63	2.01
#3	1.09	1.10	1.30	1.59	6.55	4.48	2.54	3.91
#4	1.31	0.70	1.35	1.29	7.68	4.49	4.47	1.94
#5	0.65	1.27	0.68	1.58	4.05	3.08	4.10	2.03
#6	1.04	1.13	1.02	1.11	4.15	4.09	3.19	0.94
#7	1.41	0.83	1.09	0.83	6.11	3.26	2.54	2.01
#8	0.73	0.90	0.73	0.96	3.16	4.03	1.99	1.41
#9	0.86	1.24	0.68	0.86	4.82	3.30	3.05	2.01
mean	1.00	1.01	1.03	1.21	5.09	3.47	3.01	2.01
SD	0.26	0.20	0.32	0.29	1.42	0.86	0.80	0.80
SEM	0.09	0.07	0.11	0.10	0.47	0.29	0.27	0.27

Logarithmic translation

Experiment	Control				LPS			
	Ellagic acid (µM)							
	0	10	30	100	0	10	30	100
#1	0.05	0.02	0.19	0.12	0.64	0.36	0.42	0.26
#2	-0.10	-0.07	-0.05	0.14	0.70	0.34	0.42	0.30
#3	0.04	0.04	0.11	0.20	0.82	0.65	0.40	0.59
#4	0.12	-0.16	0.13	0.11	0.89	0.65	0.65	0.29
#5	-0.19	0.10	-0.17	0.20	0.61	0.49	0.61	0.31
#6	0.02	0.05	0.01	0.05	0.62	0.61	0.50	-0.03
#7	0.15	-0.08	0.04	-0.08	0.79	0.51	0.41	0.30
#8	-0.14	-0.05	-0.14	-0.02	0.50	0.61	0.30	0.15
#9	-0.07	0.09	-0.17	-0.06	0.68	0.52	0.48	0.30
**mean**	**-0.01**	**0.00**	**0.00**	**0.07**	**0.69**	**0.53**	**0.47**	**0.28**
**SD**	**0.12**	**0.09**	**0.13**	**0.11**	**0.12**	**0.12**	**0.11**	**0.16**
**SEM**	**0.04**	**0.03**	**0.04**	**0.04**	**0.04**	**0.04**	**0.04**	**0.05**
vs. control					<0.001	<0.001	<0.001	<0.001
vs. 0 µM		0.986	0.998	0.586		0.010	<0.001	<0.001
vs. 10 µM			0.996	0.629			0.288	<0.001
vs. 30 µM				0.551				0.004

Normality Test (Shapiro-Wilk): Passed (p = 0.784)

Equal Variance Test (Brown-Forsythe): Passed (p = 0.982)

Two-Way ANOVA

Source of Variation	DF	SS	MS	F	p
LPS	1	4.116	4.116	281.684	<0.001
Dose	3	0.255	0.0851	5.824	0.001
LPS x Dose	3	0.585	0.195	13.338	<0.001
Residual	64	0.935	0.0146		
Total	71	5.891	0.0830		

Tnfa mRNA

fold vs. 0 ug/ml in control

Experiment	Control				LPS			
	Ethyl gallate (µM)							
	0	10	30	100	0	10	30	100
#1	0.93	0.92	1.09	1.08	4.55	4.96	3.02	2.99
#2	1.19	1.06	0.85	1.32	5.90	3.10	2.33	4.06
#3	0.88	0.77	1.15	1.03	6.52	5.53	5.84	2.32
#4	1.09	1.60	1.46	1.23	6.66	6.82	2.77	3.83
#5	1.00	1.39	1.40	0.86	5.75	5.51	3.49	2.65
#6	0.91	1.42	1.10	0.91	4.45	6.09	5.34	2.55
#7	1.39	1.32	1.54	1.11	6.13	6.61	3.73	3.34
#8	0.77	1.23	1.06	0.68	5.22	3.58	3.55	2.24
#9	0.85	1.49	0.99	0.97	4.71	4.15	3.71	2.59
mean	1.00	1.24	1.18	1.02	5.54	5.15	3.75	2.95
SD	0.19	0.28	0.23	0.19	0.84	1.31	1.15	0.66
SEM	0.06	0.09	0.08	0.06	0.28	0.44	0.38	0.22

Logarithmic translation

Experiment	Control				LPS			
	Ethyl gallate (µM)							
	0	10	30	100	0	10	30	100
#1	-0.03	-0.04	0.04	0.03	0.66	0.70	0.48	0.48
#2	0.07	0.02	-0.07	0.12	0.77	0.49	0.37	0.61
#3	-0.05	-0.11	0.06	0.01	0.81	0.74	0.77	0.37
#4	0.04	0.20	0.17	0.09	0.82	0.83	0.44	0.58
#5	0.00	0.14	0.15	-0.07	0.76	0.74	0.54	0.42
#6	-0.04	0.15	0.04	-0.04	0.65	0.78	0.73	0.41
#7	0.14	0.12	0.19	0.04	0.79	0.82	0.57	0.52
#8	-0.11	0.09	0.02	-0.17	0.72	0.55	0.55	0.35
#9	-0.07	0.17	0.00	-0.01	0.67	0.62	0.57	0.41
**mean**	**-0.01**	**0.08**	**0.07**	**0.00**	**0.74**	**0.70**	**0.56**	**0.46**
**SD**	**0.08**	**0.11**	**0.08**	**0.09**	**0.07**	**0.12**	**0.13**	**0.09**
**SEM**	**0.03**	**0.04**	**0.03**	**0.03**	**0.02**	**0.04**	**0.04**	**0.03**
vs. control					<0.001	<0.001	<0.001	<0.001
vs. 0 µM		0.278	0.402	0.873		0.374	<0.001	<0.001
vs. 10 µM			0.904	0.321			0.010	<0.001
vs. 30 µM				0.413				0.078

Normality Test (Shapiro-Wilk): Passed (p = 0.892)

Equal Variance Test (Brown-Forsythe): Passed (p = 0.869)

Two-Way ANOVA

Source of Variation	DF	SS	MS	F	p
LPS	1	6.014	6.014	634.514	<0.001
Dose	3	0.271	0.0904	9.534	<0.001
LPS x Dose	3	0.228	0.0759	8.010	<0.001
Residual	64	0.607	0.00948		
Total	71	7.120	0.100		

Tnfa mRNA

fold vs. 0 ug/ml in control

Experiment	Control				LPS			
	Epicatechin (µM)							
	0	10	30	100	0	10	30	100
#1	1.29	1.18	1.80	1.40	5.42	5.49	5.24	3.51
#2	0.84	1.60	1.53	1.45	4.16	3.74	4.11	2.14
#3	0.87	1.41	1.65	1.86	6.79	3.51	3.30	2.92
#4	1.13	1.73	1.51	1.52	4.86	5.82	6.13	4.73
#5	1.06	1.41	1.60	1.06	5.39	6.92	5.87	4.12
#6	0.80	1.80	1.25	1.31	6.27	5.34	4.77	3.79
#7	1.56	0.89	1.38	1.36	6.99	3.24	5.48	3.14
#8	0.94	1.06	1.32	1.04	5.27	6.46	3.71	2.59
#9	0.51	1.24	1.31	1.20	4.26	4.73	6.29	4.40
mean	1.00	1.37	1.48	1.35	5.49	5.03	4.99	3.48
SD	0.30	0.31	0.18	0.25	1.02	1.32	1.08	0.86
SEM	0.10	0.10	0.06	0.08	0.34	0.44	0.36	0.29

Logarithmic translation

Experiment	Control				LPS			
	Epicatechin (µM)							
	0	10	30	100	0	10	30	100
#1	0.11	0.07	0.26	0.15	0.73	0.74	0.72	0.55
#2	-0.07	0.20	0.18	0.16	0.62	0.57	0.61	0.33
#3	-0.06	0.15	0.22	0.27	0.83	0.55	0.52	0.46
#4	0.05	0.24	0.18	0.18	0.69	0.76	0.79	0.67
#5	0.03	0.15	0.20	0.02	0.73	0.84	0.77	0.61
#6	-0.09	0.25	0.10	0.12	0.80	0.73	0.68	0.58
#7	0.19	-0.05	0.14	0.13	0.84	0.51	0.74	0.50
#8	-0.03	0.02	0.12	0.02	0.72	0.81	0.57	0.41
#9	-0.29	0.09	0.12	0.08	0.63	0.68	0.80	0.64
**mean**	**-0.02**	**0.13**	**0.17**	**0.13**	**0.73**	**0.69**	**0.69**	**0.53**
**SD**	**0.14**	**0.10**	**0.05**	**0.08**	**0.08**	**0.12**	**0.10**	**0.11**
**SEM**	**0.05**	**0.03**	**0.02**	**0.03**	**0.03**	**0.04**	**0.03**	**0.04**
vs. control					<0.001	<0.001	<0.001	<0.001
vs. 0 µM		0.018	0.001	0.014		0.718	0.581	<0.001
vs. 10 µM			0.613	0.996			0.987	0.006
vs. 30 µM				0.757				0.007

Normality Test (Shapiro-Wilk): Passed (p = 0.606)

Equal Variance Test (Brown-Forsythe): Passed (p = 0.359)

Two-Way ANOVA

Source of Variation	DF	SS	MS	F	p
LPS	1	5.631	5.631	547.829	<0.001
Dose	3	0.114	0.0379	3.688	0.016
LPS x Dose	3	0.282	0.0941	9.159	<0.001
Residual	64	0.658	0.0103		
Total	71	6.685	0.0942		

Il1b mRNA

fold vs. 0 ug/ml in control

Experiment	Control				LPS			
	Ellagic acid (μM)							
	0	10	30	100	0	10	30	100
#1	0.9	2.2	1.6	1.1	73.4	59.9	66.4	31.2
#2	1.1	2.2	1.9	2.2	86.5	70.8	41.6	49.3
#3	1.0	2.1	1.8	2.3	43.5	56.6	33.3	30.5
#4	1.2	1.7	2.5	2.7	70.9	74.2	53.6	50.9
#5	1.0	2.1	2.4	2.6	57.4	62.8	48.2	46.7
#6	0.8	2.8	1.4	2.4	71.6	68.8	43.6	27.6
#7	1.0	1.6	2.2	1.8	59.5	59.3	48.3	30.5
#8	1.1	1.6	1.3	2.5	67.5	62.7	30.2	43.6
#9	0.9	1.3	1.1	2.1	71.1	86.1	39.7	30.3
mean	1.0	1.9	1.8	2.2	66.8	66.8	45.0	37.8
SD	0.1	0.4	0.5	0.5	12.1	9.3	10.9	9.5
SEM	0.0	0.1	0.2	0.2	4.0	3.1	3.6	3.2

Logarithmic translation

Experiment	Control				LPS			
	Ellagic acid (μM)							
	0	10	30	100	0	10	30	100
#1	-0.03	0.34	0.21	0.04	1.87	1.78	1.82	1.49
#2	0.03	0.34	0.28	0.34	1.94	1.85	1.62	1.69
#3	0.00	0.32	0.26	0.37	1.64	1.75	1.52	1.48
#4	0.08	0.23	0.40	0.43	1.85	1.87	1.73	1.71
#5	0.00	0.31	0.38	0.41	1.76	1.80	1.68	1.67
#6	-0.09	0.44	0.14	0.37	1.86	1.84	1.64	1.44
#7	0.01	0.20	0.33	0.26	1.77	1.77	1.68	1.48
#8	0.04	0.22	0.12	0.39	1.83	1.80	1.48	1.64
#9	-0.06	0.12	0.03	0.31	1.85	1.93	1.60	1.48
**mean**	**0.00**	**0.28**	**0.24**	**0.33**	**1.82**	**1.82**	**1.64**	**1.57**
**SD**	**0.05**	**0.10**	**0.13**	**0.12**	**0.09**	**0.06**	**0.10**	**0.11**
**SEM**	**0.02**	**0.03**	**0.04**	**0.04**	**0.03**	**0.02**	**0.03**	**0.04**
vs. control					<0.001	<0.001	<0.001	<0.001
vs. 0 μM		<0.001	<0.001	<0.001		0.941	<0.001	<0.001
vs. 10 μM			0.382	0.539			<0.001	<0.001
vs. 30 μM				0.181				0.190

Normality Test (Shapiro-Wilk): Passed (p = 0.299)
Equal Variance Test (Brown-Forsythe): Passed (p = 0.453)
Two-Way ANOVA

Source of Variation	DF	SS	MS	*F*	*p*
LPS	1	40.581	40.581	4319.660	<0.001
Dose	3	0.207	0.0689	7.336	<0.001
LPS x Dose	3	0.815	0.272	28.929	<0.001
Residual	64	0.601	0.00939		
Total	71	42.204	0.594		

Il1b mRNA

fold vs. 0 ug/ml in control

Experiment	Control				LPS			
	Ethyl gallate (μM)							
	0	10	30	100	0	10	30	100
#1	0.9	0.9	1.1	1.1	50.9	23.1	23.5	11.0
#2	1.0	0.7	0.9	0.9	68.6	49.2	22.1	13.2
#3	1.2	0.7	1.0	0.7	71.5	44.1	22.5	22.0
#4	1.0	1.0	1.0	1.5	69.9	32.0	14.9	13.4
#5	1.0	1.3	1.3	2.0	64.5	51.8	35.0	18.1
#6	1.0	1.1	1.1	1.4	60.1	33.8	27.9	20.7
#7	1.0	0.8	0.9	1.4	72.1	43.5	25.1	18.4
#8	1.0	0.9	0.9	1.2	65.8	44.1	24.6	16.9
#9	0.9	0.8	0.8	1.3	59.4	45.2	23.9	16.8
mean	1.0	0.9	1.0	1.3	64.8	40.8	24.4	16.7
SD	0.1	0.2	0.1	0.4	6.9	9.2	5.3	3.6
SEM	0.0	0.1	0.0	0.1	2.3	3.1	1.8	1.2

Logarithmic translation

Experiment	Control				LPS			
	Ethyl gallate (μM)							
	0	10	30	100	0	10	30	100
#1	-0.05	-0.05	0.06	0.03	1.71	1.36	1.37	1.04
#2	-0.02	-0.16	-0.07	-0.06	1.84	1.69	1.34	1.12
#3	0.06	-0.14	-0.02	-0.13	1.85	1.64	1.35	1.34
#4	0.00	-0.01	0.02	0.18	1.84	1.51	1.17	1.13
#5	-0.01	0.11	0.10	0.29	1.81	1.71	1.54	1.26
#6	0.01	0.02	0.05	0.16	1.78	1.53	1.45	1.32
#7	0.01	-0.08	-0.05	0.15	1.86	1.64	1.40	1.27
#8	0.02	-0.02	-0.03	0.09	1.82	1.64	1.39	1.23
#9	-0.03	-0.10	-0.08	0.11	1.77	1.66	1.38	1.22
**mean**	**0.00**	**-0.05**	**0.00**	**0.09**	**1.81**	**1.60**	**1.38**	**1.21**
**SD**	**0.03**	**0.08**	**0.06**	**0.13**	**0.05**	**0.11**	**0.10**	**0.10**
**SEM**	**0.01**	**0.03**	**0.02**	**0.04**	**0.02**	**0.04**	**0.03**	**0.03**
vs. control					<0.001	<0.001	<0.001	<0.001
vs. 0 μM		0.611	0.974	0.118		<0.001	<0.001	<0.001
vs. 10 μM			0.488	0.009			<0.001	<0.001
vs. 30 μM				0.134				<0.001

Normality Test (Shapiro-Wilk): Passed (p = 0.066)
Equal Variance Test (Brown-Forsythe): Passed (p = 0.211)
Two-Way ANOVA

Source of Variation	DF	SS	MS	*F*	*p*
LPS	1	39.948	39.948	5084.276	<0.001
Dose	3	0.678	0.226	28.744	<0.001
LPS x Dose	3	1.232	0.411	52.276	<0.001
Residual	64	0.503	0.00786		
Total	71	42.360	0.597		

Il1b mRNA

fold vs. 0 ug/ml in control

Experiment	Control				LPS			
	Epicatechin (μM)							
	0	10	30	100	0	10	30	100
#1	0.9	1.6	1.9	2.6	87.8	42.4	48.1	30.6
#2	0.8	1.5	1.7	3.9	61.2	37.6	29.2	32.7
#3	1.3	1.7	1.9	2.7	43.4	51.7	57.7	33.8
#4	1.1	1.6	1.8	3.2	59.9	53.6	50.7	34.5
#5	1.0	1.7	1.5	3.1	49.6	59.0	36.1	18.9
#6	0.8	1.0	1.4	2.7	85.3	64.0	36.1	24.0
#7	1.2	1.4	1.6	3.9	75.8	76.4	53.1	26.6
#8	0.9	1.8	1.7	2.0	66.5	40.1	43.7	32.2
#9	0.8	1.4	1.8	4.5	59.2	57.6	53.2	35.0
mean	1.0	1.5	1.7	3.2	65.4	53.6	45.3	29.8
SD	0.2	0.2	0.2	0.8	15.1	12.5	9.7	5.5
SEM	0.1	0.1	0.1	0.3	5.0	4.2	3.2	1.8

Logarithmic translation

Experiment	Control				LPS			
	Epicatechin (μM)							
	0	10	30	100	0	10	30	100
#1	-0.07	0.19	0.28	0.41	1.94	1.63	1.68	1.49
#2	-0.07	0.18	0.22	0.59	1.79	1.57	1.47	1.51
#3	0.11	0.24	0.28	0.43	1.64	1.71	1.76	1.53
#4	0.06	0.21	0.25	0.50	1.78	1.73	1.70	1.54
#5	0.01	0.22	0.19	0.49	1.70	1.77	1.56	1.28
#6	-0.07	0.02	0.13	0.43	1.93	1.81	1.56	1.38
#7	0.09	0.15	0.21	0.59	1.88	1.88	1.73	1.42
#8	-0.04	0.25	0.23	0.31	1.82	1.60	1.64	1.51
#9	-0.07	0.13	0.25	0.65	1.77	1.76	1.73	1.54
**mean**	**-0.01**	**0.18**	**0.23**	**0.49**	**1.81**	**1.72**	**1.65**	**1.47**
**SD**	**0.08**	**0.07**	**0.05**	**0.11**	**0.10**	**0.10**	**0.10**	**0.09**
**SEM**	**0.03**	**0.02**	**0.02**	**0.04**	**0.03**	**0.03**	**0.03**	**0.03**
vs. control					<0.001	<0.001	<0.001	<0.001
vs. 0 μM		<0.001	<0.001	<0.001		0.084	<0.001	<0.001
vs. 10 μM			0.252	<0.001			0.090	<0.001
vs. 30 μM				<0.001				<0.001

Normality Test (Shapiro-Wilk): Passed (p = 0.270)
Equal Variance Test (Brown-Forsythe): Passed (p = 0.500)
Two-Way ANOVA

Source of Variation	DF	SS	MS	*F*	*p*
LPS	1	37.195	37.195	4728.432	<0.001
Dose	3	0.0566	0.0189	2.397	0.076
LPS x Dose	3	1.630	0.543	69.053	<0.001
Residual	64	0.503	0.00787		
Total	71	39.385	0.555		

Il6 mRNA

fold vs. 0 ug/ml in control

Experiment	Control				LPS			
Ellagic acid (μM)	0	10	30	100	0	10	30	100
#1	1.1	2.2	3.6	5.7	356.6	253.8	235.4	184.7
#2	0.7	1.6	2.8	4.4	361.8	360.2	338.5	199.8
#3	1.2	1.3	3.2	4.0	362.3	274.2	213.5	214.8
#4	0.8	2.2	2.9	5.7	442.4	279.5	248.9	239.2
#5	1.2	2.1	3.0	5.0	341.7	320.3	351.6	182.5
#6	1.0	2.4	2.8	5.1	296.8	303.6	292.2	210.8
#7	1.1	2.1	2.9	3.0	327.5	230.1	374.9	254.2
#8	0.9	2.0	2.8	4.4	348.4	322.2	221.0	211.5
#9	1.0	1.8	3.1	3.7	404.5	378.4	309.8	260.3
mean	1.0	2.0	3.0	4.5	360.2	302.5	287.3	217.5
SD	0.2	0.3	0.3	0.9	42.3	48.4	60.2	28.2
SEM	0.1	0.1	0.1	0.3	14.1	16.1	20.1	9.4

Logarithmic translation

Experiment	Control				LPS			
Ellagic acid (μM)	0	10	30	100	0	10	30	100
#1	0.05	0.33	0.55	0.75	2.55	2.40	2.37	2.27
#2	-0.16	0.21	0.44	0.65	2.56	2.56	2.53	2.30
#3	0.08	0.13	0.50	0.60	2.56	2.44	2.33	2.33
#4	-0.10	0.35	0.46	0.76	2.65	2.45	2.40	2.38
#5	0.09	0.33	0.48	0.70	2.53	2.51	2.55	2.26
#6	-0.01	0.37	0.45	0.70	2.47	2.48	2.47	2.32
#7	0.05	0.31	0.47	0.47	2.52	2.36	2.57	2.41
#8	-0.04	0.30	0.45	0.64	2.54	2.51	2.34	2.33
#9	-0.01	0.26	0.49	0.57	2.61	2.58	2.49	2.42
**mean**	**-0.01**	**0.29**	**0.48**	**0.65**	**2.55**	**2.48**	**2.45**	**2.33**
**SD**	**0.08**	**0.08**	**0.03**	**0.09**	**0.05**	**0.07**	**0.09**	**0.06**
**SEM**	**0.03**	**0.03**	**0.01**	**0.03**	**0.02**	**0.02**	**0.03**	**0.02**
vs. control					<0.001	<0.001	<0.001	<0.001
vs. 0 μM		<0.001	<0.001	<0.001		0.049	0.010	<0.001
vs. 10 μM			<0.001	<0.001			0.449	<0.001
vs. 30 μM				<0.001				0.005

Normality Test (Shapiro-Wilk): Passed (p = 0.182)

Equal Variance Test (Brown-Forsythe): Passed (p = 0.072)

Two-Way ANOVA

Source of Variation	DF	SS	MS	F	p
LPS	1	79.511	79.511	15200.554	<0.001
Dose	3	0.518	0.173	32.994	<0.001
LPS x Dose	3	1.839	0.613	117.185	<0.001
Residual	64	0.335	0.00523		
Total	71	82.202	1.158		

Il6 mRNA

fold vs. 0 ug/ml in control

Experiment	Control				LPS			
Ethyl gallate (μM)	0	10	30	100	0	10	30	100
#1	1.0	1.2	3.3	8.2	366.9	225.0	120.6	59.5
#2	1.1	1.3	2.7	7.9	419.1	262.6	131.6	77.6
#3	0.9	1.3	2.8	6.8	292.3	242.5	112.9	68.1
#4	1.1	1.2	2.3	6.0	278.5	164.1	127.3	83.1
#5	0.9	1.4	2.9	6.1	441.8	221.6	145.7	74.7
#6	1.0	1.0	1.7	6.1	362.3	201.1	137.9	92.6
#7	1.2	1.3	2.0	7.5	438.0	216.5	105.5	54.6
#8	1.1	1.3	3.2	7.2	325.7	256.1	108.9	53.4
#9	0.8	1.2	2.5	6.4	306.4	247.1	107.1	54.2
mean	1.0	1.3	2.6	6.9	359.0	226.3	121.9	68.6
SD	0.1	0.1	0.5	0.8	62.8	30.6	14.5	14.2
SEM	0.0	0.0	0.2	0.3	20.9	10.2	4.8	4.7

Logarithmic translation

Experiment	Control				LPS			
Ethyl gallate (μM)	0	10	30	100	0	10	30	100
#1	0.00	0.09	0.52	0.92	2.56	2.35	2.08	1.77
#2	0.05	0.12	0.42	0.90	2.62	2.42	2.12	1.89
#3	-0.06	0.11	0.45	0.83	2.47	2.38	2.05	1.83
#4	0.03	0.08	0.36	0.78	2.44	2.22	2.10	1.92
#5	-0.04	0.13	0.46	0.78	2.65	2.35	2.16	1.87
#6	0.01	0.02	0.24	0.78	2.56	2.30	2.14	1.97
#7	0.07	0.10	0.30	0.87	2.64	2.34	2.02	1.74
#8	0.03	0.13	0.50	0.86	2.51	2.41	2.04	1.73
#9	-0.12	0.09	0.40	0.81	2.49	2.39	2.03	1.73
mean	0.00	0.10	0.40	0.84	2.55	2.35	2.08	1.83
SD	0.06	0.03	0.09	0.05	0.08	0.06	0.05	0.09
SEM	0.02	0.01	0.03	0.02	0.03	0.02	0.02	0.03
**median**	**0.01**	**0.10**	**0.42**	**0.83**	**2.56**	**2.35**	**2.08**	**1.83**
**Q1**	**-0.04**	**0.09**	**0.36**	**0.78**	**2.49**	**2.34**	**2.04**	**1.74**
**Q3**	**0.03**	**0.12**	**0.46**	**0.87**	**2.62**	**2.39**	**2.12**	**1.89**
vs. control					<0.001	<0.001	<0.001	<0.001
vs. 0 μM		0.540	<0.001	<0.001		<0.001	<0.001	<0.001
vs. 10 μM			<0.001	<0.001			<0.001	<0.001
vs. 30 μM				<0.001				<0.001

Normality Test (Shapiro-Wilk): Passed (p = 0.626)

Equal Variance Test (Brown-Forsythe): Failed (p < 0.050)

Two-factor linear model with heteroscedasticity-robust standard errors

Source of Variation	DF	F	p
LPS	1	5507.88	<0.001
Dose	3	428.33	<0.001
LPS x Dose	3	393.23	<0.001

Il6 mRNA

fold vs. 0 ug/ml in control

Experiment	Control				LPS			
Epicatechin (μM)	0	10	30	100	0	10	30	100
#1	0.8	0.8	2.0	2.8	408.3	290.4	226.5	160.2
#2	1.0	0.7	1.5	3.5	344.1	210.9	172.4	141.6
#3	1.2	0.9	2.4	3.0	316.3	237.0	224.7	100.1
#4	0.8	0.9	1.5	3.5	459.8	250.4	309.1	190.9
#5	1.0	1.2	2.8	5.2	274.7	168.2	214.9	168.6
#6	1.2	1.0	1.9	4.0	327.1	232.3	231.0	106.9
#7	1.3	1.2	2.1	3.9	304.0	329.2	254.5	126.6
#8	0.7	1.2	2.4	4.0	388.6	243.4	207.4	163.6
#9	1.0	1.1	2.4	4.0	385.1	292.1	245.5	148.3
mean	1.0	1.0	2.1	3.8	356.5	250.4	231.8	145.2
SD	0.2	0.2	0.4	0.7	58.4	48.0	37.3	29.7
SEM	0.1	0.1	0.1	0.2	19.5	16.0	12.4	9.9

Logarithmic translation

Experiment	Control				LPS			
Epicatechin (μM)	0	10	30	100	0	10	30	100
#1	-0.11	-0.11	0.30	0.45	2.61	2.46	2.36	2.20
#2	0.01	-0.15	0.18	0.54	2.54	2.32	2.24	2.15
#3	0.08	-0.05	0.37	0.48	2.50	2.37	2.35	2.00
#4	-0.11	-0.05	0.18	0.55	2.66	2.40	2.49	2.28
#5	0.00	0.06	0.45	0.72	2.44	2.23	2.33	2.23
#6	0.08	0.01	0.27	0.60	2.51	2.37	2.36	2.03
#7	0.11	0.07	0.32	0.59	2.48	2.52	2.41	2.10
#8	-0.15	0.07	0.38	0.60	2.59	2.39	2.32	2.21
#9	0.00	0.05	0.39	0.60	2.59	2.47	2.39	2.17
**mean**	**-0.01**	**-0.01**	**0.32**	**0.57**	**2.55**	**2.39**	**2.36**	**2.15**
**SD**	**0.09**	**0.08**	**0.09**	**0.08**	**0.07**	**0.09**	**0.07**	**0.09**
**SEM**	**0.03**	**0.03**	**0.03**	**0.03**	**0.02**	**0.03**	**0.02**	**0.03**
vs. control					<0.001	<0.001	<0.001	<0.001
vs. 0 μM		0.951	<0.001	<0.001		<0.001	<0.001	<0.001
vs. 10 μM			<0.001	<0.001			0.436	<0.001
vs. 30 μM				<0.001				<0.001

Normality Test (Shapiro-Wilk): Passed (p = 0.106)

Equal Variance Test (Brown-Forsythe): Passed (p = 0.898)

Two-Way ANOVA

Source of Variation	DF	SS	MS	F	p
LPS	1	82.919	82.919	11719.022	<0.001
Dose	3	0.323	0.108	15.206	<0.001
LPS x Dose	3	2.521	0.840	118.786	<0.001
Residual	64	0.453	0.00708		
Total	71	86.216	1.214		

CORRELATION

Values

			NO production		*Nos2* mRNA		*Ptgs2* mRNA		*Tnfa* mRNA		*Il1b* mRNA		*Il6* mRNA		*Il10* mRNA	
			mean	SEM	mean	SEM	mean	SEM	mean	SEM	mean	SEM	mean	SEM	mean	SEM
**Control**	**RBE**	**0 μg/ml**	0.3	0.0	1.0	0.0	1.0	0.1	1.00	0.06	1.0	0.1	1.0	0.1	1.0	0.1
**Control**	**RBE**	**50 μg/ml**	0.5	0.1	1.8	0.1	0.9	0.1	1.12	0.07	0.9	0.1	0.9	0.1	0.9	0.1
**Control**	**RBE**	**100 μg/ml**	1.4	0.2	2.0	0.2	1.0	0.1	1.08	0.06	0.9	0.1	1.0	0.0	1.1	0.0
**LPS**	**RBE**	**0 μg/ml**	59.4	0.4	101.5	6.5	24.9	2.3	5.36	0.51	66.8	6.3	363.5	25.3	11.6	0.9
**LPS**	**RBE**	**50 μg/ml**	42.1	0.9	63.4	2.6	5.6	1.0	3.81	0.40	6.6	0.4	159.2	10.2	9.2	0.7
**LPS**	**RBE**	**100 μg/ml**	25.5	0.9	20.4	1.2	3.6	1.0	2.21	0.16	1.3	0.2	71.2	6.1	9.8	0.3
**Control**	**Ellagic acid**	**0 μM**	0.3	0.1	1.0	0.1	1.0	0.1	1.00	0.09	1.0	0.0	1.0	0.1		
**Control**	**Ellagic acid**	**10 μM**	0.3	0.1	1.1	0.1	1.2	0.1	1.01	0.07	1.9	0.1	2.0	0.1		
**Control**	**Ellagic acid**	**30 μM**	0.3	0.1	0.9	0.1	2.3	0.3	1.03	0.11	1.8	0.2	3.0	0.1		
**Control**	**Ellagic acid**	**100 μM**	1.0	0.0	1.5	0.1	3.4	0.3	1.21	0.10	2.2	0.2	4.5	0.3		
**LPS**	**Ellagic acid**	**0 μM**	54.4	1.1	109.1	4.6	27.4	1.3	5.09	0.47	66.8	4.0	360.2	14.1		
**LPS**	**Ellagic acid**	**10 μM**	49.3	0.8	83.8	4.9	23.4	1.4	3.47	0.29	66.8	3.1	302.5	16.1		
**LPS**	**Ellagic acid**	**30 μM**	34.2	2.5	74.4	3.7	18.7	0.9	3.01	0.27	45.0	3.6	287.3	20.1		
**LPS**	**Ellagic acid**	**100 μM**	20.9	1.4	36.8	3.6	9.2	0.9	2.01	0.27	37.8	3.2	217.5	9.4		
**Control**	**Ethyl gallate**	**0 μM**	0.2	0.0	1.0	0.1	1.0	0.1	1.00	0.06	1.0	0.0	1.0	0.0		
**Control**	**Ethyl gallate**	**10 μM**	0.2	0.0	0.9	0.1	2.1	0.2	1.24	0.09	0.9	0.1	1.3	0.0		
**Control**	**Ethyl gallate**	**30 μM**	0.2	0.1	0.8	0.0	3.3	0.3	1.18	0.08	1.0	0.0	2.6	0.2		
**Control**	**Ethyl gallate**	**100 μM**	0.6	0.1	1.8	0.1	3.2	0.4	1.02	0.06	1.3	0.1	6.9	0.3		
**LPS**	**Ethyl gallate**	**0 μM**	56.5	2.4	103.4	4.6	23.1	1.2	5.54	0.28	64.8	2.3	359.0	20.9		
**LPS**	**Ethyl gallate**	**10 μM**	46.8	0.7	79.0	3.8	14.5	1.1	5.15	0.44	40.8	3.1	226.3	10.2		
**LPS**	**Ethyl gallate**	**30 μM**	30.9	1.8	50.0	3.7	10.7	0.7	3.75	0.38	24.4	1.8	121.9	4.8		
**LPS**	**Ethyl gallate**	**100 μM**	12.2	1.1	23.3	1.2	5.1	0.6	2.95	0.22	16.7	1.2	68.6	4.7		
**Control**	**Epicatechin**	**0 μM**	0.3	0.0	1.0	0.1	1.0	0.1	1.00	0.10	1.0	0.1	1.0	0.1		
**Control**	**Epicatechin**	**10 μM**	0.3	0.1	1.0	0.1	1.8	0.2	1.37	0.10	1.5	0.1	1.0	0.1		
**Control**	**Epicatechin**	**30 μM**	0.3	0.0	1.2	0.1	1.9	0.2	1.48	0.06	1.7	0.1	2.1	0.1		
**Control**	**Epicatechin**	**100 μM**	0.9	0.2	1.6	0.1	1.8	0.2	1.35	0.08	3.2	0.3	3.8	0.2		
**LPS**	**Epicatechin**	**0 μM**	53.3	5.4	101.3	5.3	26.5	0.9	5.49	0.34	65.4	5.0	356.5	19.5		
**LPS**	**Epicatechin**	**10 μM**	47.4	6.4	103.4	4.7	21.4	1.7	5.03	0.44	53.6	4.2	250.4	16.0		
**LPS**	**Epicatechin**	**30 μM**	41.3	7.5	99.8	4.9	16.5	1.3	4.99	0.36	45.3	3.2	231.8	12.4		
**LPS**	**Epicatechin**	**100 μM**	21.4	4.3	63.2	5.3	12.1	1.3	3.48	0.29	29.8	1.8	145.2	9.9		

Logarithmic transformation

			NO production				*Ptgs2* mRNA		*Tnfa* mRNA						*Il10* mRNA	
			mean	SEM			mean	SEM	mean	SEM					mean	SEM
**Control**	**RBE**	**0 μg/ml**	-0.52	0.05			0.00	0.03	0.00	0.03					0.00	0.03
**Control**	**RBE**	**50 μg/ml**	-0.29	0.05			-0.07	0.03	0.05	0.03					-0.06	0.04
**Control**	**RBE**	**100 μg/ml**	0.15	0.05			-0.02	0.03	0.03	0.03					0.03	0.02
**LPS**	**RBE**	**0 μg/ml**	1.77	0.00			1.40	0.04	0.73	0.04					1.06	0.03
**LPS**	**RBE**	**50 μg/ml**	1.62	0.01			0.75	0.08	0.58	0.04					0.95	0.04
**LPS**	**RBE**	**100 μg/ml**	1.41	0.02			0.56	0.14	0.34	0.03					0.99	0.02
**Control**	**Ellagic acid**	**0 μM**	-0.47	0.07			0.00	0.05	0.00	0.04						
**Control**	**Ellagic acid**	**10 μM**	-0.57	0.10			0.09	0.03	0.00	0.03						
**Control**	**Ellagic acid**	**30 μM**	-0.59	0.11			0.37	0.05	0.01	0.04						
**Control**	**Ellagic acid**	**100 μM**	-0.01	0.02			0.53	0.04	0.08	0.04						
**LPS**	**Ellagic acid**	**0 μM**	1.74	0.01			1.44	0.02	0.71	0.04						
**LPS**	**Ellagic acid**	**10 μM**	1.69	0.01			1.37	0.03	0.54	0.04						
**LPS**	**Ellagic acid**	**30 μM**	1.53	0.03			1.27	0.02	0.48	0.04						
**LPS**	**Ellagic acid**	**100 μM**	1.32	0.03			0.97	0.04	0.30	0.05						
**Control**	**Ethyl gallate**	**0 μM**	-0.64	0.03			0.00	0.06	0.00	0.03						
**Control**	**Ethyl gallate**	**10 μM**	-0.75	0.08			0.32	0.04	0.09	0.04						
**Control**	**Ethyl gallate**	**30 μM**	-0.67	0.11			0.52	0.04	0.07	0.03						
**Control**	**Ethyl gallate**	**100 μM**	-0.24	0.04			0.51	0.05	0.01	0.03						
**LPS**	**Ethyl gallate**	**0 μM**	1.75	0.02			1.36	0.02	0.74	0.02						
**LPS**	**Ethyl gallate**	**10 μM**	1.67	0.01			1.16	0.03	0.71	0.04						
**LPS**	**Ethyl gallate**	**30 μM**	1.49	0.03			1.03	0.03	0.57	0.04						
**LPS**	**Ethyl gallate**	**100 μM**	1.09	0.04			0.71	0.05	0.47	0.03						
**Control**	**Epicatechin**	**0 μM**	-0.46	0.04			0.00	0.04	0.00	0.05						
**Control**	**Epicatechin**	**10 μM**	-0.60	0.14			0.26	0.05	0.14	0.03						
**Control**	**Epicatechin**	**30 μM**	-0.55	0.04			0.29	0.05	0.17	0.02						
**Control**	**Epicatechin**	**100 μM**	-0.04	0.08			0.26	0.05	0.13	0.03						
**LPS**	**Epicatechin**	**0 μM**	1.73	0.05			1.42	0.01	0.74	0.03						
**LPS**	**Epicatechin**	**10 μM**	1.68	0.07			1.33	0.04	0.70	0.04						
**LPS**	**Epicatechin**	**30 μM**	1.62	0.09			1.22	0.03	0.70	0.03						
**LPS**	**Epicatechin**	**100 μM**	1.33	0.10			1.08	0.05	0.54	0.04						

**Normality Test (Shapiro-Wilk):**	Failed ($p \le 0.001$)	Passed ($p = 0.244$)	Passed ($p = 0.129$)	Failed ($p \le 0.001$)	Failed ($p = 0.002$)	Passed ($p = 0.550$)
**Constant Variance Test (Spearman Rank Correlation):**	Failed ($p \le 0.001$)	Passed ($p = 0.331$)	Passed ($p = 0.255$)	Failed ($p \le 0.001$)	Failed ($p \le 0.001$)	Passed ($p = 0.060$)
**Spearman Rank Correlation:**	$\rho = 0.964$			$\rho = 0.833$	$\rho = 0.871$	
	$p < 0.001$			$p < 0.001$	$p < 0.001$	
**Pearson Correlation:**		$r = 0.918$	$r = 0.942$			$r = 0.978$
		$p < 0.001$	$p < 0.001$			$p < 0.001$

NO PRODUCTION

uM nitrite

Experiment	RBE (µg/mL) 0	50	100	Ethyl acetate (µg/mL) 0	50	100	Pure butanol (µg/mL) 0	50	100
#1	0.13	0.74	1.31	0.13	0.21	1.41	0.13	0.92	1.78
#2	0.29	1.04	1.23	0.29	0.21	1.59	0.29	0.78	1.65
#3	0.24	0.67	1.36	0.24	0.29	1.64	0.24	0.91	1.77
#4	0.08	0.36	1.34	0.08	0.22	1.38	0.08	1.17	1.82
#5	0.13	1.05	1.29	0.13	0.20	1.83	0.13	1.33	1.64
#6	0.26	0.54	1.41	0.49	0.29	1.56	0.49	1.02	1.76
**mean**	0.19	0.73	1.32	**0.23**	**0.23**	**1.57**	**0.23**	**1.02**	**1.74**
**SD**	0.09	0.27	0.06	**0.15**	**0.04**	**0.16**	**0.15**	**0.20**	**0.08**
**SEM**	0.04	0.11	0.03	**0.06**	**0.02**	**0.07**	**0.06**	**0.08**	**0.03**
**median**	**0.19**	**0.71**	**1.33**						
**Q1**	**0.13**	**0.57**	**1.29**						
**Q3**	**0.26**	**0.97**	**1.36**						
vs. 0 µg/ml		0.002	<0.001		0.916	<0.001		<0.001	<0.001
vs. 50 µg/ml			0.002						<0.001

Normality Test (Shapiro-Wilk) for RBE: Passed (p = 0.270)
Equal Variance Test (Brown-Forsythe) for RBE: Failed (p < 0.050)
Welch's One-Way ANOVA

Source of Variation	DF	*F*	*p*
Between groups	2	57.4	<0.001

Normality Test (Shapiro-Wilk) for ethyl acetate: Passed (p = 0.110)
Equal Variance Test (Brown-Forsythe) for ethyl acetate: Passed (p = 0.145)
One-Way ANOVA

Source of Variation	DF	SS	MS	*F*	*p*
Between groups	2	7.160	3.580	206.183	<0.001
Residual	15	0.260	0.0174		.
Total	17	7.420			

Normality Test (Shapiro-Wilk) for pure butanol: Passed (p = 0.273)
Equal Variance Test (Brown-Forsythe) for pure butanol: Passed (p = 0.248)
One-Way ANOVA

Source of Variation	DF	SS	MS	*F*	*p*
Between groups	2	6.838	3.419	149.781	<0.001
Residual	15	0.342	0.0228		.
Total	17	7.180			

NO PRODUCTION

uM nitrite

Experiment	Ellagic acid (µM) 0	10	30	100	Ethyl gallate (µM) 0	10	30	100	Epicatechin (µM) 0	10	30	100
#1	0.20	0.15	0.23	4.60	0.18	0.41	0.36	0.27	0.19	0.06	0.01	0.14
#2	0.41	0.04	0.06	4.36	0.21	0.39	0.16	0.21	0.18	0.05	0.22	0.33
#3	0.27	0.30	0.22	4.52	0.24	0.48	0.54	0.40	0.16	0.12	0.21	0.27
#4	0.31	0.52	0.66	2.37	0.25	0.20	0.38	0.32	0.20	0.35	0.08	0.29
#5	0.32	0.28	0.65	2.85	0.18	0.21	0.24	0.25	0.28	0.14	0.18	0.25
#6	0.33	0.07	0.51	2.98	0.27	0.47	0.30	0.20	0.40	0.20	0.12	0.39
#7	0.21	0.13	0.19	2.58	0.35	0.40	0.15	0.03	0.18	0.43	0.33	0.23
#8	0.15	0.13	0.20	2.54	0.35	0.36	0.46	0.62	0.33	0.26	0.35	0.43
#9	0.28	0.32	0.08	2.25	0.30	0.41	0.50	0.53	0.16	0.38	0.28	0.38
**mean**	0.27	0.21	0.31	3.23	**0.26**	**0.37**	**0.34**	**0.32**	**0.23**	**0.22**	**0.20**	**0.30**
**SD**	0.08	0.15	0.23	0.98	**0.07**	**0.10**	**0.14**	**0.18**	**0.08**	**0.14**	**0.11**	**0.09**
**SEM**	0.03	0.05	0.08	0.33	**0.02**	**0.03**	**0.05**	**0.06**	**0.03**	**0.05**	**0.04**	**0.03**
vs. 0 uM						0.374	0.616	0.832		0.867	0.896	0.565

Logarithmic translation

Experiment	Ellagic acid 0	10	30	100
#1	-0.70	-0.83	-0.63	0.66
#2	-0.38	-1.42	-1.20	0.64
#3	-0.57	-0.52	-0.66	0.65
#4	-0.52	-0.29	-0.18	0.37
#5	-0.50	-0.55	-0.19	0.45
#6	-0.48	-1.19	-0.29	0.47
#7	-0.68	-0.88	-0.72	0.41
#8	-0.82	-0.90	-0.70	0.41
#9	-0.56	-0.50	-1.08	0.35
**mean**	-0.58	-0.79	-0.63	0.49
**SD**	0.13	0.36	0.36	0.13
**SEM**	0.04	0.12	0.12	0.04
**median**	**-0.54**	**-0.71**	**-0.50**	**0.41**
**Q1**	**-0.65**	**-0.90**	**-0.71**	**0.38**
**Q3**	**-0.50**	**-0.51**	**-0.21**	**0.44**
vs. 0 uM		0.408	0.712	<0.001

Normality Test (Shapiro-Wilk) for RBE: Passed (p > 0.050)
Equal Variance Test (Brown-Forsythe) for RBE: Failed (p < 0.050)
Welch's One-Way ANOVA

Source of Variation	DF	*F*	*p*
Between groups	3	41.54	<0.001

Normality Test (Shapiro-Wilk) for ethyl gallate: Passed (p = 0.992)
Equal Variance Test (Brown-Forsythe) for ethyl gallate: Passed (p = 0.115)
One-Way ANOVA

Source of Variation	DF	SS	MS	*F*	*p*
Between groups	3	0.0620	0.0207	1.236	0.313
Residual	32	0.535	0.0167		
Total	35	0.596			

Normality Test (Shapiro-Wilk) for epicatechin: Passed (p = 0.434)
Equal Variance Test (Brown-Forsythe) for epicatechin: Passed (p = 0.190)
One-Way ANOVA

Source of Variation	DF	SS	MS	*F*	*p*
Between groups	3	0.0522	0.0174	1.447	0.248
Residual	32	0.385	0.0120		
Total	35	0.437			

CELL VIABILITY

%

Experiment	DEA NONOate (μM)					
	0	1	3	10	30	100
#1	108.5	115.8	93.2	102.2	111.7	106.3
#2	106.8	112.3	103.4	98.6	102.1	115.1
#3	81.9	105.4	103.2	101.4	109.5	109.2
#4	102.8	112.9	112.5	97.0	107.5	115.5
#5	97.8	113.6	110.8	104.7	104.8	104.0
#6	89.8	104.7	104.0	102.0	94.0	101.0
#7	100.6	104.3	100.0	102.0	88.6	106.9
#8	111.8	98.9	103.5	94.7	90.2	101.3
**mean**	**100.0**	**108.5**	**103.8**	**100.3**	**101.1**	**107.4**
**SD**	**10.0**	**5.9**	**6.0**	**3.3**	**9.0**	**5.6**
**SEM**	**3.5**	**2.1**	**2.1**	**1.2**	**3.2**	**2.0**
vs. 0 μM		0.094	0.627	0.921	0.945	0.150

Normality Test (Shapiro-Wilk): Passed (p = 0.461)

Equal Variance Test (Brown-Forsythe): Passed (p = 0.132)

One-Way ANOVA

Source of Variation	DF	SS	MS	*F*	*p*
Between Groups	5	549.811	109.96	2.241	0.068
Residual	42	2061.147	49.075		
Total	47	2610.958			

CELL VIABILITY

%

Experiment	GSNO (μM)					
	0	3	10	30	100	300
#1	99.8	104.5	108.1	102.6	102.6	83.4
#2	92.1	105.5	95.3	106.0	102.0	82.1
#3	107.4	101.5	103.9	99.9	97.0	74.8
#4	100.6	103.6	98.0	108.6	111.3	77.3
#5	115.1	114.1	102.4	96.2	107.9	83.2
#6	102.3	98.1	99.7	94.3	102.4	84.6
#7	93.0	96.3	97.1	97.5	108.1	87.1
#8	89.7	97.6	92.8	111.4	104.7	84.9
**mean**	**100.0**	**102.6**	**99.7**	**102.1**	**104.5**	**82.2**
**SD**	**8.5**	**5.7**	**5.0**	**6.2**	**4.5**	**4.1**
**SEM**	**3.0**	**2.0**	**1.8**	**2.2**	**1.6**	**1.5**
vs. 0 uM		0.751	0.912	0.732	0.430	<0.001

Normality Test (Shapiro-Wilk): Passed (p = 0.505)

Equal Variance Test (Brown-Forsythe): Passed (p = 0.407)

One-Way ANOVA

Source of Variation	DF	SS	MS	*F*	*p*
Between Groups	5	2685.942	537.19	15.736	<0.001
Residual	42	1433.800	34.138		
Total	47	4119.742			

NO PRODUCTION

uM nitrite

Experiment	Control					LPS				
	DEA NONOate (μM)									
	0	1	3	10	30	0	1	3	10	30
#1	0.3	1.6	3.7	12.9	39.3	56.8	55.6	52.8	61.2	76.7
#2	0.2	1.4	3.8	12.8	36.8	58.3	56.4	53.1	63.9	77.9
#3	0.3	1.5	3.8	12.9	38.5	56.7	56.8	54.8	65.6	82.7
#4	0.2	1.2	3.5	13.0	38.6	56.0	55.6	53.0	53.4	81.2
#5	0.3	1.1	3.3	12.9	36.9	54.1	57.4	54.5	62.8	84.1
#6	0.3	1.2	3.4	12.9	33.8	57.9	60.7	57.7	66.2	86.8
mean	0.3	1.3	3.6	12.9	37.3	56.6	57.1	54.3	62.2	81.6
SD	0.1	0.2	0.2	0.1	2.0	1.5	1.9	1.8	4.7	3.8
SEM	0.0	0.1	0.1	0.0	0.8	0.6	0.8	0.8	1.9	1.6
**median**	**0.3**	**1.3**	**3.6**	**12.9**	**37.7**	**56.8**	**56.6**	**53.8**	**63.3**	**82.0**
**Q1**	**0.2**	**1.2**	**3.4**	**12.9**	**36.8**	**56.1**	**55.8**	**53.0**	**61.6**	**78.7**
**Q3**	**0.3**	**1.5**	**3.8**	**12.9**	**38.6**	**57.6**	**57.3**	**54.8**	**65.2**	**83.7**
vs. control						<0.001	<0.001	<0.001	<0.001	<0.001
vs. 0 μM		0.004	<0.001	<0.001	<0.001		0.936	0.068	0.002	<0.001

Normality Test (Shapiro-Wilk): Failed (p < 0.050)

Kruskal-Wallis Test: H = 56.593 with 9 degrees of freedom (p =< 0.001)

NO PRODUCTION

uM nitrite

Experiment	Control					LPS				
	GSNO (μM)									
	0	3	10	30	100	0	3	10	30	100
#1	0.4	0.8	2.5	7.3	15.4	50.0	54.0	55.2	55.3	55.0
#2	0.3	0.8	2.3	7.3	20.0	50.3	54.5	53.7	55.7	60.5
#3	0.3	1.0	2.2	7.1	13.9	54.6	55.5	54.6	53.0	56.2
#4	0.3	0.8	2.4	6.8	17.3	52.5	56.6	54.4	55.5	55.9
#5	0.3	0.6	2.2	6.9	22.2	53.9	57.3	55.3	57.1	62.1
#6	0.4	0.9	2.3	7.0	22.3	53.8	56.6	55.1	56.8	62.2
mean	0.3	0.8	2.3	7.1	18.5	52.5	55.7	54.7	55.6	58.6
SD	0.1	0.1	0.1	0.2	3.5	2.0	1.3	0.6	1.4	3.3
SEM	0.0	0.0	0.0	0.1	1.4	0.8	0.5	0.2	0.6	1.4
**median**	**0.3**	**0.8**	**2.3**	**7.1**	**18.6**	**53.2**	**56.0**	**54.9**	**55.6**	**58.3**
**Q1**	**0.3**	**0.8**	**2.2**	**7.0**	**15.8**	**50.9**	**54.7**	**54.4**	**55.4**	**56.0**
**Q3**	**0.4**	**0.9**	**2.4**	**7.2**	**21.6**	**53.9**	**56.6**	**55.2**	**56.6**	**61.7**
vs. control						<0.001	<0.001	<0.001	<0.001	0.002
vs. 0 μM		0.004	<0.001	<0.001	<0.001		<0.001	<0.001	<0.001	<0.001

Normality Test (Shapiro-Wilk): Failed (p < 0.050)

Kruskal-Wallis Test: H = 55.439 with 9 degrees of freedom (p =< 0.001)
